# Skilful precipitation nowcasting using deep generative models of radar

**DOI:** 10.1038/s41586-021-03854-z

**Published:** 2021-09-29

**Authors:** Suman Ravuri, Karel Lenc, Matthew Willson, Dmitry Kangin, Remi Lam, Piotr Mirowski, Megan Fitzsimons, Maria Athanassiadou, Sheleem Kashem, Sam Madge, Rachel Prudden, Amol Mandhane, Aidan Clark, Andrew Brock, Karen Simonyan, Raia Hadsell, Niall Robinson, Ellen Clancy, Alberto Arribas, Shakir Mohamed

**Affiliations:** 1 DeepMind, London, UK; 2 Met Office, Exeter, UK; 3 University of Exeter, Exeter, UK; 4 University of Reading, Reading, UK

**Keywords:** Environmental sciences, Computer science

## Abstract

Precipitation nowcasting, the high-resolution forecasting of precipitation up to two hours ahead, supports the real-world socioeconomic needs of many sectors reliant on weather-dependent decision-making^1,2^. State-of-the-art operational nowcasting methods typically advect precipitation fields with radar-based wind estimates, and struggle to capture important non-linear events such as convective initiations^3,4^. Recently introduced deep learning methods use radar to directly predict future rain rates, free of physical constraints^5,6^. While they accurately predict low-intensity rainfall, their operational utility is limited because their lack of constraints produces blurry nowcasts at longer lead times, yielding poor performance on rarer medium-to-heavy rain events. Here we present a deep generative model for the probabilistic nowcasting of precipitation from radar that addresses these challenges. Using statistical, economic and cognitive measures, we show that our method provides improved forecast quality, forecast consistency and forecast value. Our model produces realistic and spatiotemporally consistent predictions over regions up to 1,536 km × 1,280 km and with lead times from 5–90 min ahead. Using a systematic evaluation by more than 50 expert meteorologists, we show that our generative model ranked first for its accuracy and usefulness in 89% of cases against two competitive methods. When verified quantitatively, these nowcasts are skillful without resorting to blurring. We show that generative nowcasting can provide probabilistic predictions that improve forecast value and support operational utility, and at resolutions and lead times where alternative methods struggle.

## Abstract (web summary)

A deep generative model using radar observations is used to create skilful precipitation predictions that are accurate and support real-world utility.

## Main

The high-resolution forecasting of rainfall and hydrometeors zero to two hours into the future, known as precipitation nowcasting, is crucial for weather-dependent decision-making. Nowcasting informs the operations of a wide variety of sectors, including emergency services, energy management, retail, flood early-warning systems, air traffic control and marine services^1,2^. For nowcasting to be useful in these applications the forecast must provide accurate predictions across multiple spatial and temporal scales, account for uncertainty and be verified probabilistically, and perform well on heavier precipitation events that are rarer, but more critically affect human life and economy.

Ensemble numerical weather prediction (NWP) systems, which simulate coupled physical equations of the atmosphere to generate multiple realistic precipitation forecasts, are natural candidates for nowcasting as one can derive probabilistic forecasts and uncertainty estimates from the ensemble of future predictions^7^. For precipitation at zero to two hours lead time, NWPs tend to provide poor forecasts as this is less than the time needed for model spin-up and due to difficulties in non-Gaussian data assimilation^8–10^. As a result, alternative methods that make predictions using composite radar observations have been used; radar data is now available (in the UK) every five minutes and at 1 km × 1 km grid resolution^11^. Established probabilistic nowcasting methods, such as STEPS and PySTEPS^3,4^, follow the NWP approach of using ensembles to account for uncertainty, but model precipitation following the advection equation with a radar source term. In these models, motion fields are estimated by optical flow, smoothness penalties are used to approximate an advection forecast, and stochastic perturbations are added to the motion field and intensity model^3,4,12^. These stochastic simulations allow for ensemble nowcasts from which both probabilistic and deterministic forecasts can be derived and are applicable and consistent at multiple spatial scales, from the kilometre scale to the size of a catchment area^13^.

Approaches based on deep learning have been developed that move beyond reliance on the advection equation^5,6,14–19^. By training these models on large corpora of radar observations rather than relying on in-built physical assumptions, deep learning methods aim to better model traditionally difficult non-linear precipitation phenomena, such as convective initiation and heavy precipitation. This class of methods directly predicts precipitation rates at each grid location, and models have been developed for both deterministic and probabilistic forecasts. As a result of their direct optimization and fewer inductive biases, the forecast quality of deep learning methods—as measured by per-grid-cell metrics such as critical success index (CSI)^20^ at low precipitation levels (less than 2 mm h^−1^)—has greatly improved.

As a number of authors have noted^5,6^, forecasts issued by current deep learning systems express uncertainty at increasing lead times with blurrier precipitation fields, and may not include small-scale weather patterns that are important for improving forecast value. Furthermore, the focus in existing approaches on location-specific predictions, rather than probabilistic predictions of entire precipitation fields, limits their operational utility and usefulness, being unable to provide simultaneously consistent predictions across multiple spatial and temporal aggregations. The ability to make skilful probabilistic predictions is also known to provide greater economic and decision-making value than deterministic forecasts^21,22^.

Here we demonstrate improvements in the skill of probabilistic precipitation nowcasting that improves their value. To create these more skilful predictions, we develop an observations-driven approach for probabilistic nowcasting using deep generative models (DGMs). DGMs are statistical models that learn probability distributions of data and allow for easy generation of samples from their learned distributions. As generative models are fundamentally probabilistic, they have the ability to simulate many samples from the conditional distribution of future radar given historical radar, generating a collection of forecasts similar to ensemble methods. The ability of DGMs to both learn from observational data as well as represent uncertainty across multiple spatial and temporal scales makes them a powerful method for developing new types of operationally useful nowcasting. These models can predict smaller-scale weather phenomena that are inherently difficult to predict due to underlying stochasticity, which is a critical issue for nowcasting research. DGMs predict the location of precipitation as accurately as systems tuned to this task while preserving spatiotemporal properties useful for decision-making. Importantly, they are judged by professional meteorologists as substantially more accurate and useful than PySTEPS or other deep learning systems.

## Generative models of radar

Our nowcasting algorithm is a conditional generative model that predicts *N* future radar fields given *M* past, or contextual, radar fields, using radar-based estimates of surface precipitation **X**_T_ at a given time point *T*. Our model includes latent random vectors **Z** and parameters **θ**, described by

1 $$\begin{array}{c}P({{\bf{X}}}_{M+1{\rm{:}}M+N}|{{\bf{X}}}_{1{\rm{:}}M})=\int P({{\bf{X}}}_{M+1{\rm{:}}M+N}{\rm{|}}{\bf{Z}},{{\bf{X}}}_{1{\rm{:}}M},{\boldsymbol{\theta }})P({\bf{Z}}{\rm{|}}{{\bf{X}}}_{1{\rm{:}}M}){\rm{d}}{\bf{Z}}.\end{array}$$

**Fig. 1:**
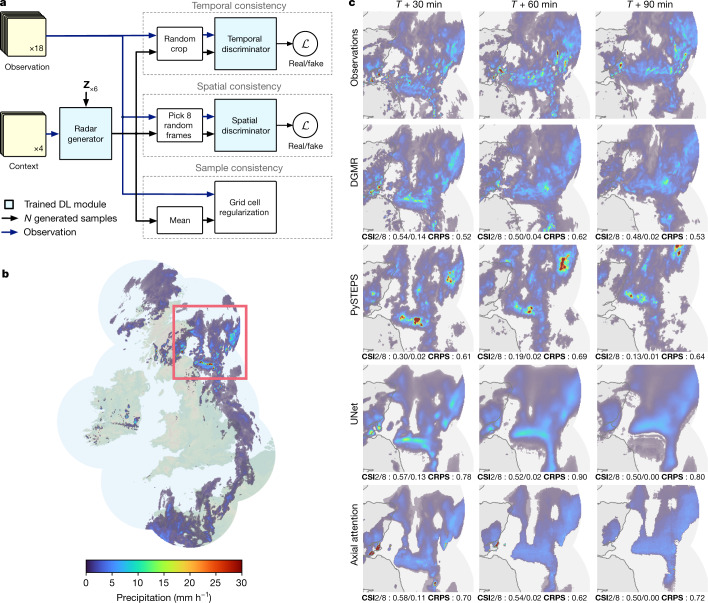
Model overview and case study of performance on a challenging precipitation event starting on = 24 June 2019 at 16:15 UK, showing convective cells over eastern Scotland. DGMR is better able to predict the spatial coverage and convection compared to other methods over a longer time period, while not over-estimating the intensities, and is significantly preferred by meteorologists (93% first choice, *n* = 56, *P* < 10^−4^). **a**, Schematic of the model architecture showing the generator with spatial latent vectors **Z**. **b**, Geographic context for the predictions. **c**, A single prediction at *T* + 30, *T* + 60 and *T* + 90 min lead time for different models. Critical success index (CSI) at thresholds 2 mm h^−1^ and 8 mm h^−1^ and continuous ranked probability score (CRPS) for an ensemble of four samples shown in the bottom left corner. For axial attention we show the mode prediction. Images are 256 km × 256 km. Maps produced with Cartopy and SRTM elevation data^46^.

The integration over latent variables ensures that the model makes predictions that are spatially dependent. Learning is framed in the algorithmic framework of a conditional generative adversarial network (GAN)^23–25^, specialized for the precipitation prediction problem. Four consecutive radar observations (the previous 20 min) are used as context for a generator (Fig. 1a) that allows sampling of multiple realizations of future precipitation, each realization being 18 frames (90 min).

Learning is driven by two loss functions and a regularization term, which guide parameter adjustment by comparing real radar observations to those generated by the model. The first loss is defined by a spatial discriminator, which is a convolutional neural network that aims to distinguish individual observed radar fields from generated fields, ensuring spatial consistency and discouraging blurry predictions. The second loss is defined by a temporal discriminator, which is a three-dimensional (3D) convolutional neural network that aims to distinguish observed and generated radar sequences, imposes temporal consistency and penalizes jumpy predictions. These two discriminators share similar architectures to existing work in video generation^26^. When used alone, these losses lead to accuracy on par with Eulerian persistence. To improve accuracy, we introduce a regularization term that penalizes deviations at the grid cell resolution between the real radar sequences and the model predictive mean (computed with multiple samples). This third term is important for the model to produce location-accurate predictions and improve performance. In the Supplementary Information, we show an ablation study supporting the necessity of each loss term. Finally, we introduce a fully convolutional latent module for the generator, allowing for predictions over precipitation fields larger than the size used at training time, while maintaining spatiotemporal consistency. We refer to this DGM of rainfall as DGMR in the text.

**Extended Data Fig. 1:**
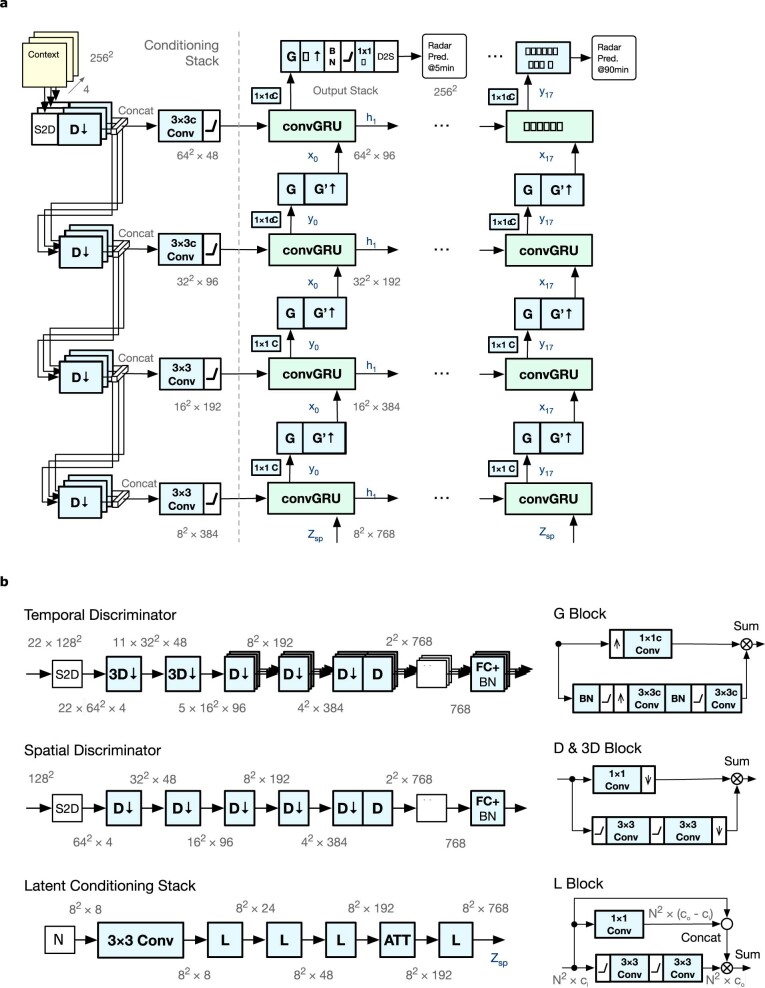
Detailed architecture of DGMR. **a**, Generator architecture. S2D is space- to-depth operation; D2S is depth to space. **b**, Temporal discriminator architecture (top left), spatial discriminator (middle left), and latent conditioning stack (bottom left) of the generator. On the right are architectures for the G block (top), D and 3D block (middle), and L block (right). For all panels, (↑) or (↓) indicates spatial up-sampling or down-sampling, respectively.

**Extended Data Fig. 2:**
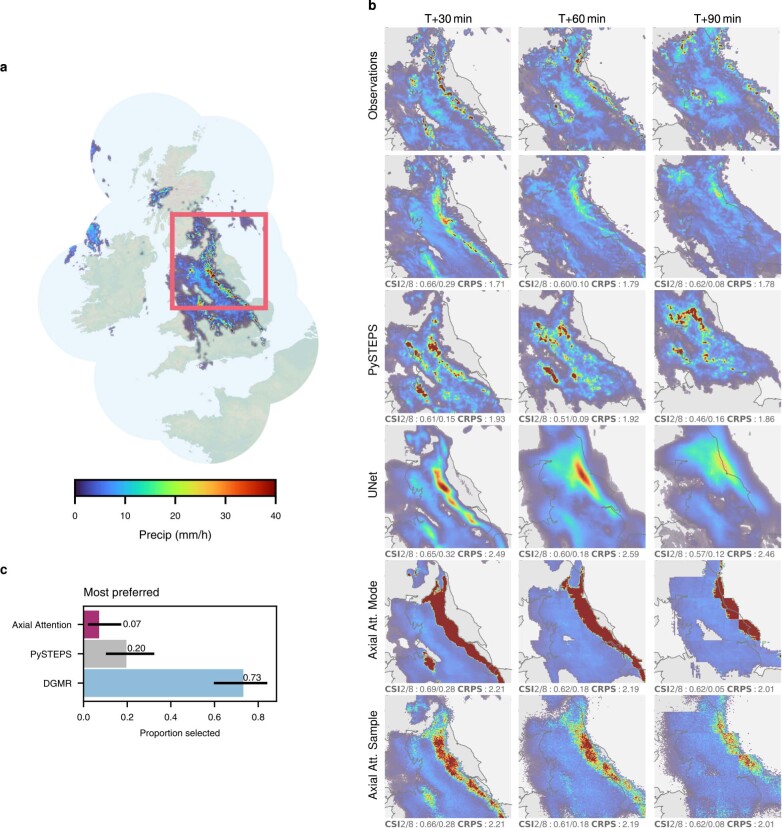
Case study of performance on a challenging precipitation event starting at 2019-07-24 at 03:15 UK, showing two separate banded structures of intense rainfall in the north-east and south-west over northern England, DGMR is better able to predict the spatial coverage and convection compared to other methods over a longer time period, while not over-estimating the intensities, and is significantly preferred by meteorologists (73% first choice, *N* = 56, *p* < 2 × 10^−4^). **a**, Geographic context for the predictions. **b**, A single prediction at *T* + 30, *T* + 60, and *T* + 90 min lead time for different models. CSI at thresholds 2 mm h^−1^and 8 mm h^−1^ and CRPS for an ensemble of four samples shown in a bottom left corner. For axial attention we show the mode prediction and the single sample. Images are 256 km × 256 km. **c**, Expert meteorologist preference for the visualized prediction (axial attention uses the mode prediction; we report the percentage of meteorologists for their first-choice rating as well as the Clopper–Pearson 95% confidence interval). Maps produced with Cartopy and SRTM elevation data^46^.

**Extended Data Fig. 3:**
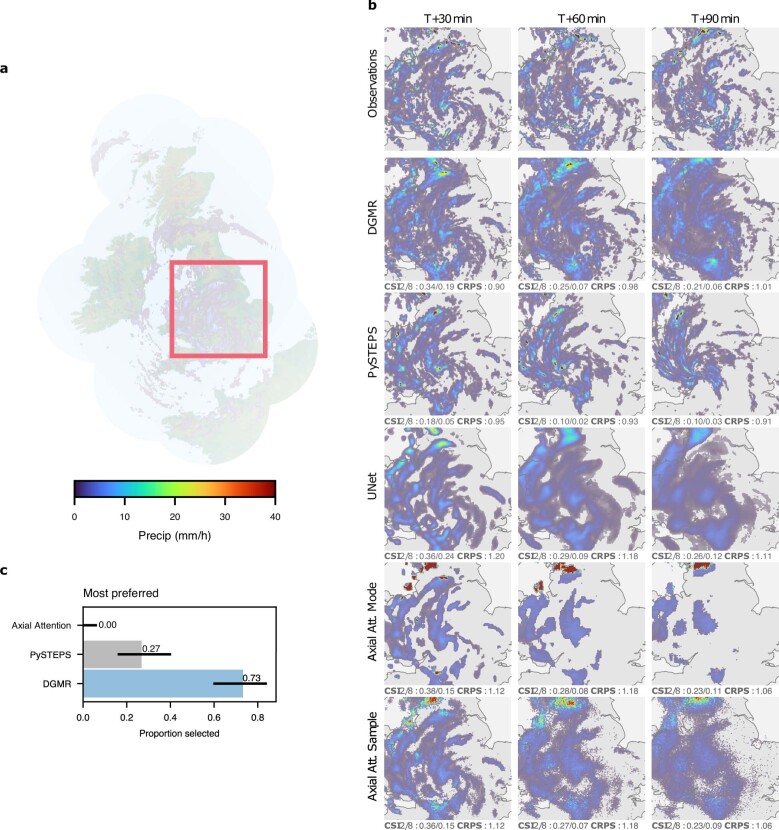
Case study of performance on a challenging precipitation event starting 2019-07-30 at 15:15 UK, showing a pattern of precipitation around a low-pressure area which is slow moving, resulting in the cyclonic banded structures over England, DGMR captures extent of precipitation overall over the area, though slightly overdoes rain coverage between bands, and is significantly preferred by meteorologists (73% first choice, *N* = 56, *p* < 2 × 10^−4^). **a**, Geographic context for the predictions. **b**, A single prediction at *T* + 30, *T* + 60, and *T* + 90 min lead time for different models. CSI at thresholds 2 mm h^−1^ and 8 mm h^−1^ and CRPS for an ensemble of four samples shown in a bottom left corner. For axial attention we show the mode prediction and the single sample. Images are 256 km × 256 km. **c**, Expert meteorologist preference for the visualized prediction (axial attention uses the mode prediction; we report the percentage of meteorologists for their first-choice rating as well as the Clopper–Pearson 95% confidence interval). Maps produced with Cartopy and SRTM elevation data^46^.

**Extended Data Fig. 4:**
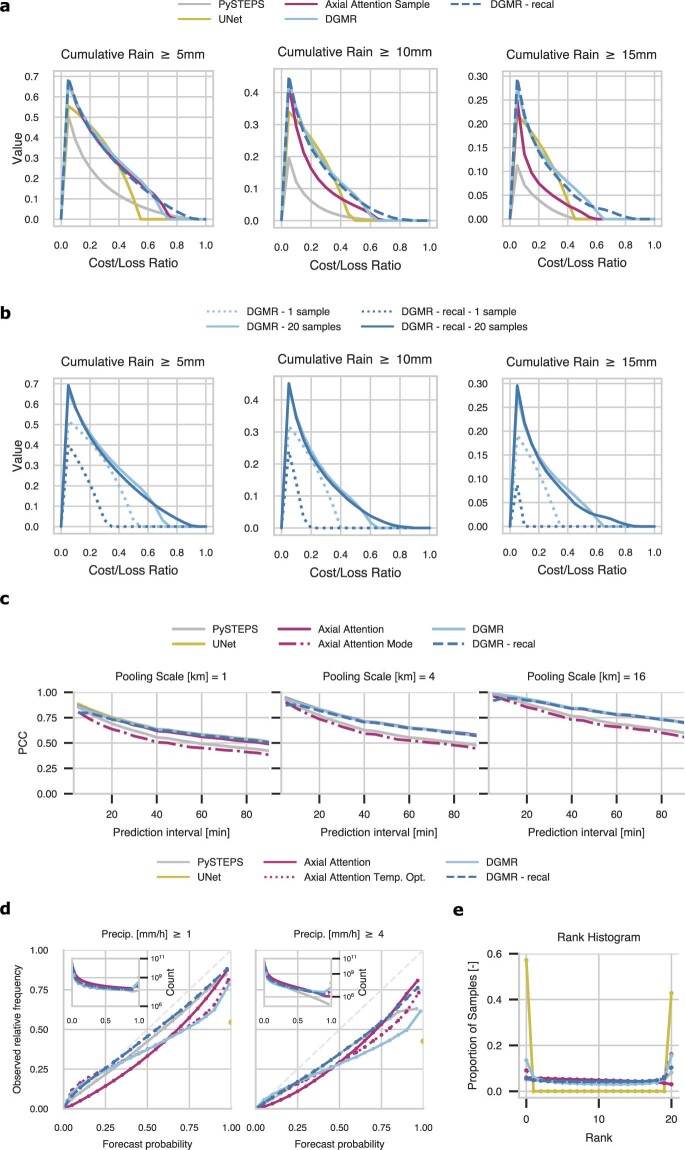
Further verification scores for the UK in 2019. **a**, Comparison of relative economic value across 20 samples of different models for different rain accumulations. UNet generates a single deterministic prediction. **b**, Effect of larger ensemble in increasing economic value. **c**, Pearson correlation coefficient of various models at grid-resolution (left), rain rates averaged over a 4 km aggregation (middle) and averaged over a 16 km aggregation (right). **d**, Reliability diagrams and sharpness plots for two precipitation thresholds for *T* + 60 min predictions. **e**, Rank histogram at *T* + 60 min.

**Extended Data Fig. 5:**
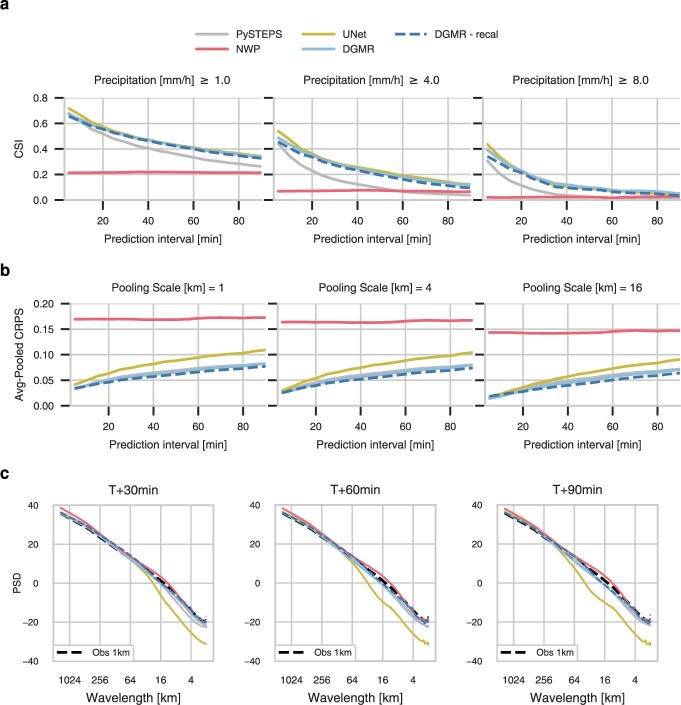
Verification scores for the UK by yearly splits aligned with NWP initialization times. **a**, CSI across 20 samples of different models across precipitation thresholds 1 mm h^−1^ (left), 4 mm h^−1^, 8 mm h^−1^ (right). UNet generates a single deterministic prediction. **b**, CRPS of various models for original predictions (left), average rain rate over a 4 km aggregation (middle), and average rain rate over a 16 km aggregation (right). **c**, Radially averaged power spectral density for full-frame 2019 predictions for different models. Please note that these results are computed with significantly fewer examples of the UK yearly dataset due to the NWP lead time alignment.

**Extended Data Fig. 6:**
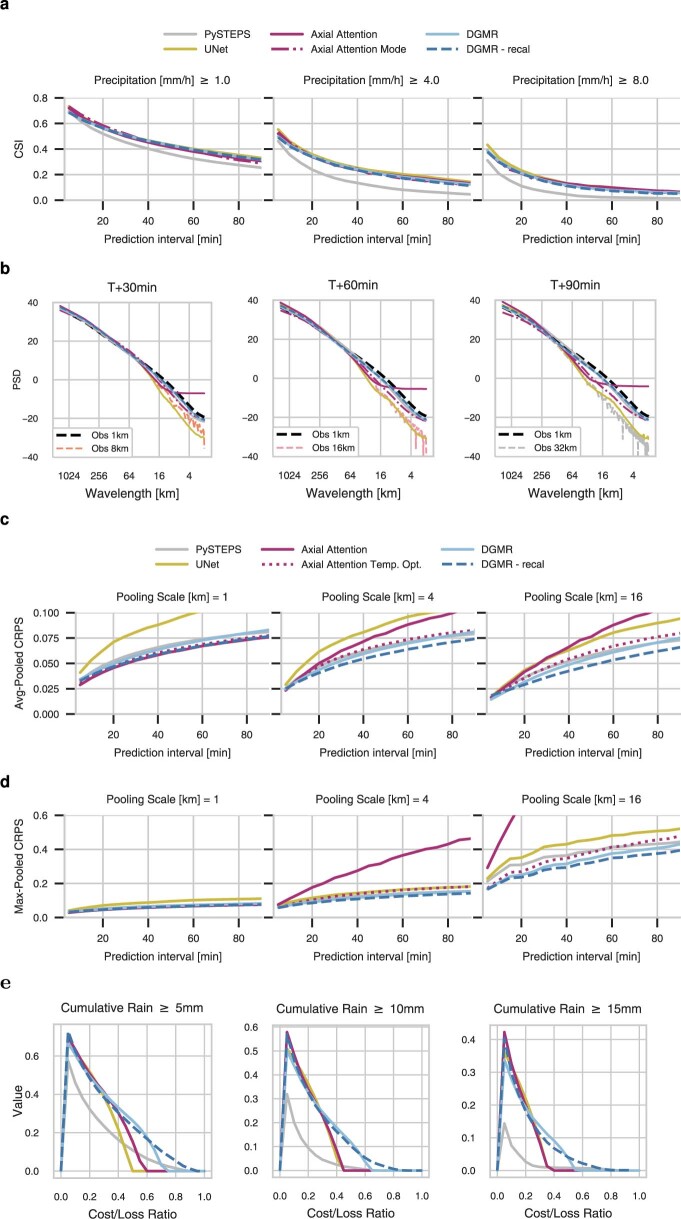
Verification scores for the UK (weekly split). **a**, CSI for 1 mm h^−1^ (left), 4 mm h^−1^, 8 mm h^−1^ (right) precipitation thresholds. **b**, Radially averaged power spectral density for full-frame predictions at *T* + 30 (left), *T* + 60 (middle), and *T* + 90 min (right). **c**, CRPS at grid-scale (left), rain rates averaged over a 4 km aggregation (middle), rain rates averaged over a 16 km aggregation (right). **d**, CRPS at grid scale (left), maximum rain rate over a 4 km aggregation (middle), and maximum rain rate over a 16 km aggregation (right). **e**, Relative economic value analysis using 20 samples for three 90 min rainfall accumulations, using 4 km aggregation. UNet generates a single deterministic prediction.

**Extended Data Fig. 7:**
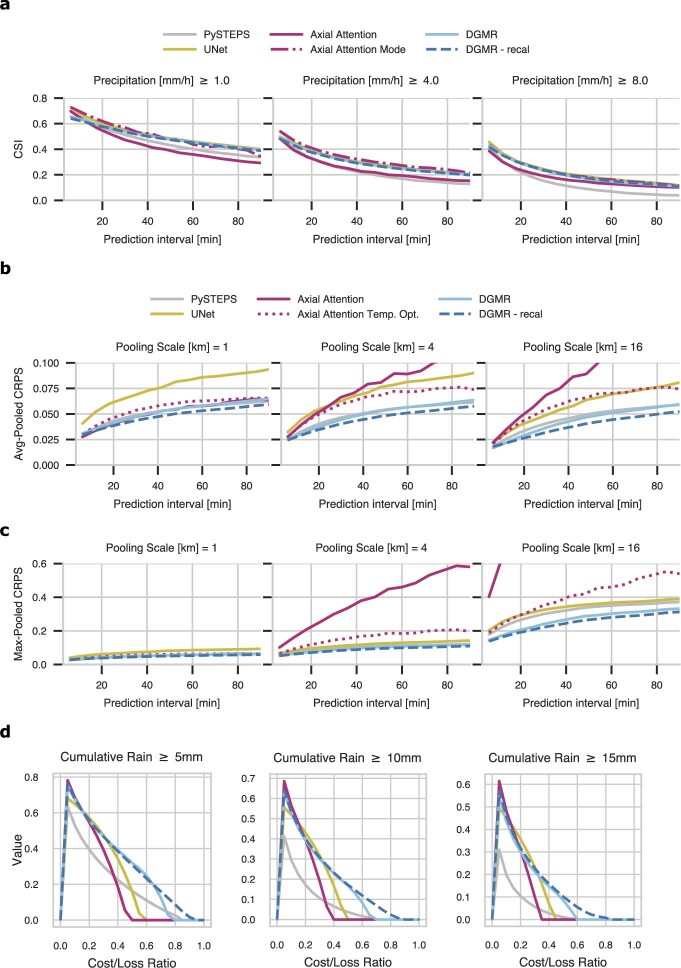
Verification scores for the United States in 2019. **a**, CSI for 1 mm h^−1^ (left), 4 mm h^−1^, 8 mm h^−1^ (right) precipitation thresholds. **b**, CRPS at grid-resolution (left), CRPS for rain rates averaged over a 4 km × 4 km area (middle), CRPS for rain rates averaged over a 16 km × 16 km area (right). **c**, CRPS at grid-resolution (left), maximum rain rate over a 4 km × 4 km area (middle), and maximum rain rate over a 16 km × 16 km area (right). **d**, Relative economic value analysis across 20 samples of different models for three 90 min rainfall accumulations, using 4 km aggregation. UNet generates a single deterministic prediction.

**Extended Data Fig. 8:**
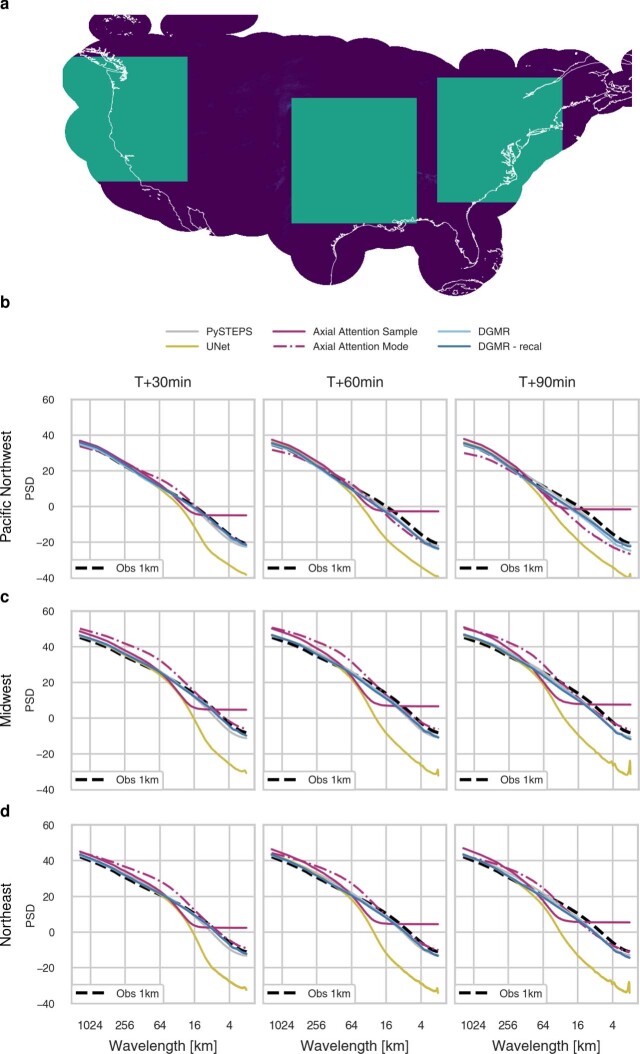
Radially averaged power spectral density for the United States in 2019. **a**, Map of United States with three 1,536 × 1,536 regions: Pacific Northwest (left), Midwest (middle), Northeast (right). **b**, Radially averaged power spectral density for Pacific Northwest region for different models at *T* + 30 (left), *T* + 60 (middle), and *T* + 90 min (right). **c**, Radially averaged power spectral density for Midwest region for different models at *T* + 30 (left), *T* + 60 (middle) and *T* + 90 min (right). **d**, Radially averaged power spectral density for Northeast region for different models at *T* + 30 (left), *T* + 60 (middle), and *T* + 90 min (right). Map produced with Cartopy.

**Extended Data Fig. 9:**
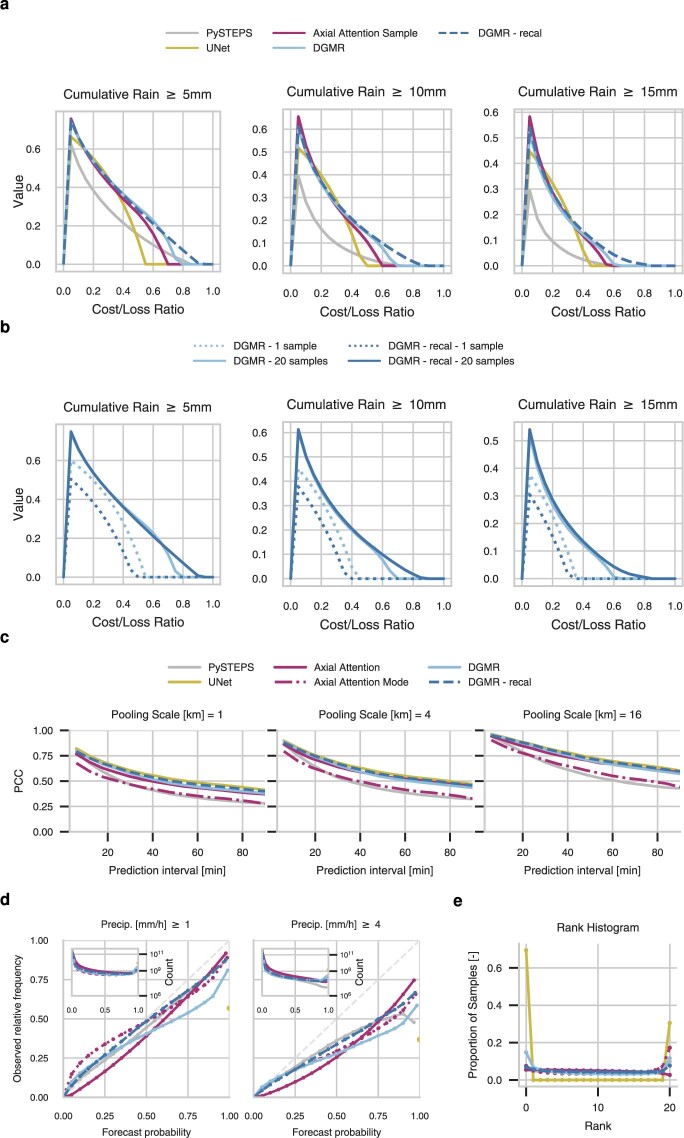
Further verification scores for the United States in 2019. **a**, Comparison of relative economic value for 20 samples for different rain accumulations. UNet generates a single deterministic prediction. **b**, Effect of larger ensemble in increasing economic value. **c**, Pearson correlation coefficient of various models at grid-resolution (left), rain rates averaged over a 4 km aggregation (middle), and rain rates averaged over a 16 km aggregation (right). **d**, Reliability diagrams and sharpness plots for two precipitation thresholds for *T* + 60 min predictions. **e**, Rank histogram at *T* + 60 min.

The model is trained on a large corpus of precipitation events, which are 256 × 256 crops extracted from the radar stream, of length 110 min (22 frames). An importance-sampling scheme is used to create a dataset more representative of heavy precipitation (Methods). Throughout, all models are trained on radar observations for the UK for years 2016–2018 and evaluated on a test set from 2019. Analysis using a weekly train–test split of the data, as well as data of the USA, is reported in Extended Data Figs. 1–9 and the Supplementary Information. Once trained, this model allows fast full-resolution nowcasts to be produced, with a single prediction (using an NVIDIA V100 GPU) needing just over a second to generate.

## Intercomparison case study

We use a single case study to compare the nowcasting performance of the generative method DGMR to three strong baselines: PySTEPS, a widely used precipitation nowcasting system based on ensembles, considered to be state-of-the-art^3,4,13^; UNet, a popular deep learning method for nowcasting^15^; and an axial attention model, a radar-only implementation of MetNet^19^. For a meteorologically challenging event, Figs. 1b, c and 4b shows the ground truth and predicted precipitation fields at *T* + 30, *T* + 60 and *T* + 90 min, quantitative scores on different verification metrics, and comparisons of expert meteorologist preferences among the competing methods. Two other cases are included in Extended Data Figs. 2 and 3.

The event in Fig. 1 shows convective cells in eastern Scotland with intense showers over land. Maintaining such cells is difficult and a traditional method such as PySTEPS overestimates the rainfall intensity over time, which is not observed in reality and does not sufficiently cover the spatial extent of the rainfall. The UNet and axial attention models roughly predict the location of rain, but owing to aggressive blurring, over-predict areas of rain, miss intensity and fail to capture any small-scale structure. By comparison, DGMR preserves a good spatial envelope, represents the convection and maintains heavy rainfall in the early prediction, although with less accurate rates at *T* + 90 min and at the edge of the radar than at previous time steps. When expert meteorologists judged these predictions against ground truth observations, they significantly preferred the generative nowcasts, with 93% of meteorologists choosing it as their first choice (Fig. 4b).

The figures also include two common verification scores. These predictions are judged as significantly different by experts, but the scores do not provide this insight. This study highlights a limitation of using existing popular metrics to evaluate forecasts: while standard metrics implicitly assume that models, such as NWPs and advection-based systems, preserve the physical plausibility of forecasts, deep learning systems may outperform on certain metrics by failing to satisfy other needed characteristics of useful predictions.

## Forecast skill evaluation

We verify the performance of competing methods using a suite of metrics as is standard practice, as no single verification score can capture all desired properties of a forecast. We report the CSI^27^ to measure location accuracy of the forecast at various rain rates. We report the radially averaged power spectral density (PSD)^28,29^ to compare the precipitation variability of nowcasts to that of the radar observations. We report the continuous ranked probability score (CRPS)^30^ to determine how well the probabilistic forecast aligns with the ground truth. For CRPS, we show pooled versions, which are scores on neighbourhood aggregations that show whether a prediction is consistent across spatial scales. Details of these metrics, and results on other standard metrics, can be found in Extended Data Figs. 1–9 and the Supplementary Information. We report results here using data from the UK, and results consistent with these showing generalization of the method on data from the USA in Extended Data Figs. 1–9.

**Fig. 2:**
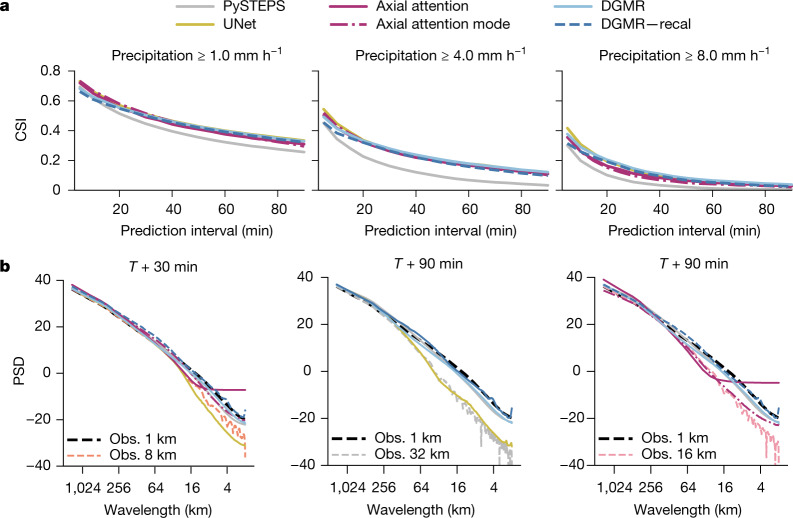
Deterministic verification scores for the UK in 2019. **a**, CSI across 20 samples for precipitation thresholds at 1 mm h^−1^ (left), 4 mm h^−1^ (middle) and 8 mm h^−1^ (right). We also report results for the axial attention mode prediction. UNet generates a single deterministic prediction. **b**, Radially averaged power spectral density for full-frame 2019 predictions for all models at *T* + 30 min (left) and *T* + 90 min (middle and right). At *T* + 90 min, UNet (middle) has an effective resolution of 32 km; both axial attention (right) sample and mode predictions have an effective resolution of 16 km. Source data.

Figure 2a shows that all three deep learning systems produce forecasts that are significantly more location-accurate than the PySTEPS baseline when compared using CSI. Using paired permutation tests with alternating weeks as independent units to assess statistical significance, we find that DGMR has significant skill compared to PySTEPS for all precipitation thresholds (*n* = 26, *P* < 10^−4^) (Methods).

The PSD in Fig. 2b shows that both DGMR and PySTEPS match the observations in their spectral characteristics, but the axial attention and UNet models produce forecasts with medium- and small-scale precipitation variability that decreases with increasing lead time. As they produce blurred predictions, the effective resolution of the axial attention and UNet nowcasts is far less than the 1 km × 1 km resolution of the data. At *T* + 90 min, the effective resolution for UNet is 32 km and for axial attention is 16 km, reducing the value of these nowcasts for meteorologists.

**Fig. 3:**
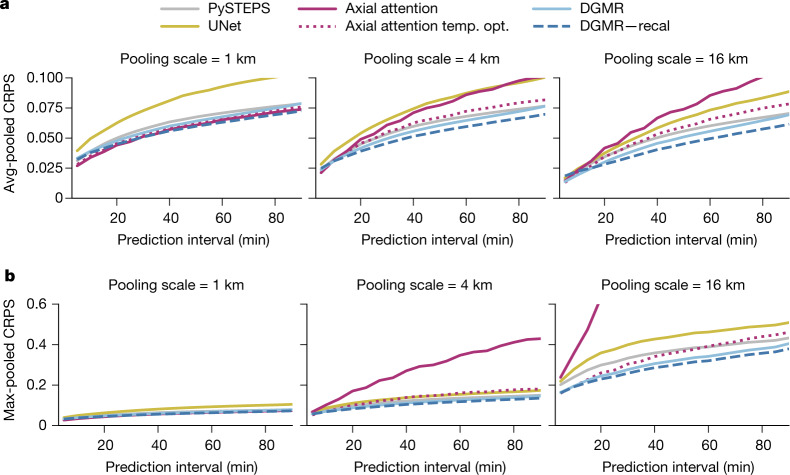
Probabilistic verification scores for the UK in 2019. Graphs show CRPS scores at the grid resolution (left), 4-km aggregations (middle) and 16-km aggregations (right). **a**, Pooled CRPS using the average rain rate. **b**, Pooled CRPS using the maximum rain rate. Source data.

For probabilistic verification, Fig. 3a, b shows the CRPS of the average and maximum precipitation rate aggregated over regions of increasing size^31^. When measured at the grid-resolution level, DGMR, PySTEPS and axial attention perform similarly; we also show an axial attention model with improved performance obtained by rescaling its output probabilities^32^ (denoted ‘axial attention temp. opt.’). As the spatial aggregation is increased, DGMR and PySTEPS provide consistently strong performance, with DGMR performing better on maximum precipitation. The axial attention model is significantly poorer for larger aggregations and underperforms all other methods at scale four and above. Using alternating weeks as independent units, paired permutation tests show that the performance differences between DGMR and the axial attention temp. opt. are significant (*n* = 26, *P* < 10^−3^).

NWP and PySTEPS methods include post-processing that is used by default in their evaluation to improve reliability. We show a simple post-processing method for DGMR in Figs. 2 and 3 (denoted ‘recal’) (Methods), which further improves its skill scores over the uncalibrated approach. Post-processing improves the reliability diagrams and rank histogram to be as or more skilful than the baseline methods (Extended Data Fig. 4). We also show evaluation on other metrics, performance on a data split over weeks rather than years, and evaluation recapitulating the inability of NWPs to make predictions at nowcasting timescales (Extended Data Figs. 4–6). We show results on a US dataset in Extended Data Figs. 7–9.

Together, these results show that the generative approach verifies competitively compared to alternatives: it outperforms (on CSI) the incumbent STEPS nowcasting approach, provides probabilistic forecasts that are more location accurate, and preserves the statistical properties of precipitation across spatial and temporal scales without blurring whereas other deep learning methods do so at the expense of them.

## Forecast value evaluation

We use both economic and cognitive analyses to show that the improved skill of DGMR results in improved decision-making value.

**Fig. 4:**
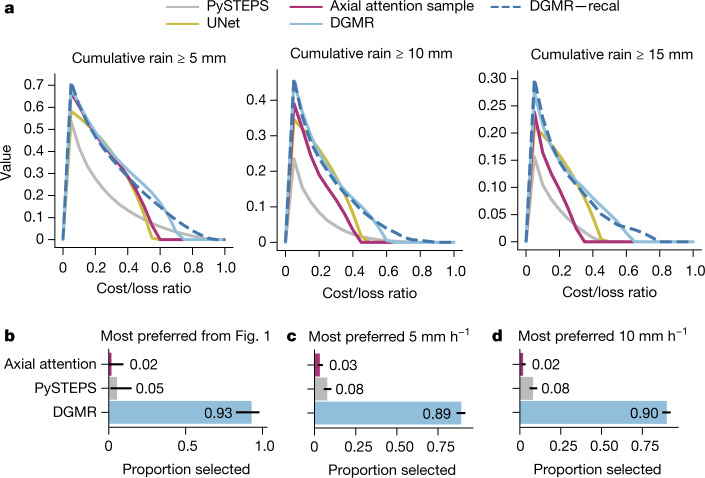
DGMR provides greater decision-making value when assessed using both economic and cognitive analyses. **a**, Relative economic value analysis across 20 samples for three 90-min rainfall accumulations, using 4-km aggregations. UNet generates a single deterministic prediction. **b**, Meteorologist preferences for the case study in Fig. 1. **c**, Meteorologist rankings for medium rain (5 mm h^−1^) cases. **d**, Meteorologist rankings for heavy rain (10 mm h^−1^) cases. Horizontal bars show the percentage of meteorologists who chose each method as their first choice. Whisker lines show the Clopper–Pearson 95% confidence interval. Meteorologists significantly preferred DGMR to alternatives (*n* = 56, *P *< 10^−4^). Source data.

We report the relative economic value of the ensemble prediction to quantitatively evaluate the benefit of probabilistic predictions using a simple and widely used decision-analytic model^22^; see the Supplementary Information for a description. Figure 4a shows that DGMR provides the highest economic value relative to the baseline methods (has highest peak and greater area under the curve). We use 20 member ensembles and show three accumulation levels used for weather warnings by Met Éireann (the Irish Meteorological service uses warnings defined directly in mm h^−1^; https://www.met.ie/weather-warnings). This analysis shows the ability of the generative ensemble to capture uncertainty, and we show the improvement with samples in Extended Data Figs. 4 and 9, and postage stamp plots to visualize the ensemble variability in Supplementary Data 1–3.

Importantly, we ground this economic evaluation by directly assessing decision-making value using the judgments of expert meteorologists working in the 24/7 operational centre at the Met Office (the UK’s national meteorology service). We conducted a two-phase experimental study to assess expert judgements of value, involving a panel of 56 experts. In phase 1, all meteorologists were asked to provide a ranked preference assessment on a set of nowcasts with the instruction that ‘preference is based on [their] opinion of accuracy and value’. Each meteorologist assessed a unique set of nowcasts, which, at the population level, allows for uncertainty characteristics and meteorologist idiosyncrasies to be averaged out in reporting the statistical effect. We randomly selected 20% of meteorologists to participate in a phase 2 retrospective recall interview^33^.

Operational meteorologists seek utility in forecasts for critical events, safety and planning guidance. Therefore, to make meaningful statements of operational usefulness, our evaluation assessed nowcasts for high-intensity events, specifically medium rain (rates above 5 mm h^−1^) and heavy rain (rates above 10 mm h^−1^). Meteorologists were asked to rank their preferences on a sample of 20 unique nowcasts (from a corpus of 2,126 events, being all high-intensity events in 2019). Data were presented in the form shown in Fig. 1b, c, showing clearly the initial context at *T* + 0 min, the ground truth at *T* + 30 min, *T* + 60 min, and *T* + 90 min, and nowcasts from PySTEPS, axial attention and DGMR. The identity of the methods in each panel was anonymized and their order randomized. See the Methods for further details of the protocol and of the ethics approval for human subjects research.

The generative nowcasting approach was significantly preferred by meteorologists when asked to make judgments of accuracy and value of the nowcast, being their most preferred 89% (95% confidence interval (CI) [0.86, 0.92]) of the time for the 5 mm h^−1^ nowcasts (Fig. 4c; *P* < 10^−4^), and 90% (95% CI [0.87, 0.92]) for the 10 mm h^−1^ nowcasts (Fig. 4d, *P* < 10^−4^). We compute the *P* value assessing the binary decision whether meteorologists chose DGMR as their first choice using a permutation test with 10,000 resamplings. We indicate the Clopper–Pearson CI. This significant meteorologist preference is important as it is strong evidence that generative nowcasting can provide meteorologists with physical insight not provided by alternative methods, and provides a grounded verification of the economic value analysis in Fig. 4a.

Meteorologists were not swayed by the visual realism of the predictions, and their responses in the subsequent structured interviews showed that they approached this task by making deliberate judgements of accuracy, location, extent, motion and rainfall intensity, and reasonable trade-offs between these factors (Supplementary Information, section C.6). In the phase 2 interviews, PySTEPS was described as “being too developmental which would be misleading”, that is, as having many “positional errors” and “much higher intensity compared with reality”. The axial attention model was described as “too bland”, that is, as being “blocky” and “unrealistic”, but had “good spatial extent”. Meteorologists described DGMR as having the “best envelope”, “representing the risk best”, as having “much higher detail compared to what [expert meteorologists] are used to at the moment”, and as capturing “both the size of convection cells and intensity the best”. In the cases where meteorologists chose PySTEPS or the axial attention as their first choice, they pointed out that DGMR showed decay in the intensity for heavy rainfall at *T* + 90 min and had difficulty predicting isolated showers, which are important future improvements for the method. See the Supplementary Information for further reports from this phase of the meteorologist assessment.

## Conclusion

Skilful nowcasting is a long-standing problem of importance for much of weather-dependent decision-making. Our approach using deep generative models directly tackles this important problem, improves on existing solutions and provides the insight needed for real-world decision-makers. We showed—using statistical, economic and cognitive measures—that our approach to generative nowcasting provides improved forecast quality, forecast consistency and forecast value, providing fast and accurate short-term predictions at lead times where existing methods struggle.

Yet, there remain challenges for our approach to probabilistic nowcasting. As the meteorologist assessment demonstrated, our generative method provides skilful predictions compared to other solutions, but the prediction of heavy precipitation at long lead times remains difficult for all approaches. Critically, our work reveals that standard verification metrics and expert judgments are not mutually indicative of value, highlighting the need for newer quantitative measurements that are better aligned with operational utility when evaluating models with few inductive biases and high capacity. Whereas existing practice focuses on quantitative improvements without concern for operational utility, we hope this work will serve as a foundation for new data, code and verification methods—as well as the greater integration of machine learning and environmental science in forecasting larger sets of environmental variables—that makes it possible to both provide competitive verification and operational utility.

## Methods

We provide additional details of the data, models and evaluation here, with references to extended data that add to the results provided in the main text.

### Datasets

A dataset of radar for the UK was used for all the experiments in the main text. Additional quantitative results on a US dataset are available in Supplementary Information section A.

### UK dataset

To train and evaluate nowcasting models over the UK, we use a collection of radar composites from the Met Office RadarNet4 network. This network comprises more than 15 operational, proprietary C-band dual polarization radars covering 99% of the UK (see figure 1 in ref. ^34^). We refer to ref. ^11^ for details about how radar reflectivity is post-processed to obtain the two-dimensional radar composite field, which includes orographic enhancement and mean field adjustment using rain gauges. Each grid cell in the 1,536 × 1,280 composite represents the surface-level precipitation rate (in mm h^−1^) over a 1 km × 1 km region in the OSGB36 coordinate system. If a precipitation rate is missing (for example, because the location is not covered by any radar, or if a radar is out of order), the corresponding grid cell is assigned a negative value which is used to mask the grid cell at training and evaluation time. The radar composites are quantized in increments of 1/32 mm h^−1^.

We use radar collected every five minutes between 1 January 2016 and 31 December 2019. We use the following data splits for model development. Fields from the first day of each month from 2016 to 2018 are assigned to the validation set. All other days from 2016 to 2018 are assigned to the training set. Finally, data from 2019 are used for the test set, preventing data leakage and testing for out of distribution generalization. For further experiments testing in-distribution performance using a different data split, see Supplementary Information section C.

### Training set preparation

Most radar composites contain little to no rain. Supplementary Table 2 shows that approximately 89% of grid cells contain no rain in the UK. Medium to heavy precipitation (using rain rate above 4 mm h^−1^) comprises fewer than 0.4% of grid cells in the dataset. To account for this imbalanced distribution, the dataset is rebalanced to include more data with heavier precipitation radar observations, which allows the models to learn useful precipitation predictions.

Each example in the dataset is a sequence of 24 radar observations of size 1,536 × 1,280, representing two continuous hours of data. The maximum rain rate is capped at 128 mm h^−1^, and sequences that are missing one or more radar observations are removed. 256 × 256 crops are extracted and an importance sampling scheme is used to reduce the number of examples containing little precipitation. We describe this importance sampling and the parameters used in Supplementary Information section A.1. After subsampling and removing entirely masked examples, the number of examples in the training set is roughly 1.5 million.

### Model details and baselines

Here, we describe the proposed method and the three baselines to which we compare performance. When applicable, we describe both the architectures of the models and the training methods. There is a wealth of prior work, and we survey them as additional background in Supplementary Information section E.

### DGMR

A high-level description of the model was given in the main text and in Fig. 1a, and we provide some insight into the design decisions here.

#### Architecture design

The nowcasting model is a generator that is trained using two discriminators and an additional regularization term. Extended Data Fig. 1 shows a detailed schematic of the generative model and the discriminators. More precise descriptions of these architectures are given in Supplement B and corresponds to the code description; pseudocode is also available in the Supplementary Information.

The generator in Fig. 1a comprises the conditioning stack which processes past four radar fields that is used as context. Making effective use of such context is typically a challenge for conditional generative models, and this stack structure allows information from the context data to be used at multiple resolutions, and is used in other competitive video GAN models, for example, in ref. ^26^. This stack produces a context representation that is used as an input to the sampler. A latent conditioning stack takes samples from *N*(0, 1) Gaussian distribution, and reshapes into a second latent representation. The sampler is a recurrent network formed with convolutional gated recurrent units (GRUs) that uses the context and latent representations as inputs. The sampler makes predictions of 18 future radar fields (the next 90 min). This architecture is both memory efficient and has had success in other forecasting applications. We also made comparisons with longer context using the past 6 or 8 frames, but this did not result in appreciable improvements.

Two discriminators in Fig. 1b are used to allow for adversarial learning in space and time. The spatial and temporal discriminator share the same structure, except that the temporal discriminator uses 3D convolutions to account for the time dimension. Only 8 out of 18 lead times are used in the spatial discriminator, and a random 128 × 128 crop used for the temporal discriminator. These choices allow the models to fit within memory. We include a spatial attention block in the latent conditioning stack since it allows the model to be more robust across different types of regions and events, and provides an implicit regularization to prevent overfitting, particularly for the US dataset.

Both the generator and discriminators use spectrally normalized convolutions throughout, similar to ref. ^35^, since this is widely established to improve optimization. During model development, we initially found that including a batch normalization layer (without variance scaling) prior to the linear layer of the two discriminators improved training stability. The results presented use batch normalization, but we later were able to obtain nearly identical quantitative and qualitative results without it.

#### Objective function

The generator is trained with losses from the two discriminators and a grid cell regularization term (denoted ${{\mathscr{L}}}_{{\rm{R}}}(\theta )$). The spatial discriminator *D*_*ϕ*_ has parameters *ϕ*, the temporal discriminator *T*_*ψ*_ has parameters *ψ*, and the generator *G*_**θ**_ has parameters **θ**. We indicate the concatenation of two fields using the notation {**X**; *G*} . The generator objective that is maximized is

2 $$\begin{array}{c}{{\mathscr{L}}}_{{\rm{G}}}({\boldsymbol{\theta }})={{\mathbb{E}}}_{{{\bf{X}}}_{1{\rm{:}}M+N}}[{{\mathbb{E}}}_{{\bf{Z}}}[D({G}_{{\boldsymbol{\theta }}}({\bf{Z}}{\rm{;}}{{\bf{X}}}_{1{\rm{:}}M}))+T({\rm{\{}}{{\bf{X}}}_{1{\rm{:}}M}{\rm{;}}{G}_{{\boldsymbol{\theta }}}({\bf{Z}}{\rm{;}}{{\bf{X}}}_{1{\rm{:}}M}){\rm{\}}})]-\lambda {{\mathscr{L}}}_{R}({\boldsymbol{\theta }})]{\rm{;}}\end{array}$$

3 $$\begin{array}{c}{{\mathscr{L}}}_{{\rm{R}}}({\boldsymbol{\theta }})=\frac{1}{{HWN}}{{\rm{|}}|({{\mathbb{E}}}_{{\bf{Z}}}[{G}_{{\boldsymbol{\theta }}}({\bf{Z}}{\rm{;}}{{\bf{X}}}_{1{\rm{:}}M})]-{{\bf{X}}}_{M+1{\rm{:}}M+N}])\odot w({{\bf{X}}}_{M+1{\rm{:}}M+N})|{\rm{|}}}_{1}.\end{array}$$

We use Monte Carlo estimates for expectations over the latent **Z** in equations (2) and (3). These are calculated using six samples per input **X**_1:*M*_, which comprises *M* = 4 radar observations. The grid cell regularizer ensures that the mean prediction remains close to the ground truth, and is averaged across all grid cells along the height *H*, width *W* and lead-time *N* axes. It is weighted towards heavier rainfall targets using the function *w*(*y*) = max(*y* + 1,  24), which operate element-wise for input vectors, and is clipped at 24 for robustness to spuriously large values in the radar. The GAN spatial discriminator loss ${{\mathscr{L}}}_{{\rm{D}}}(\phi )$ and temporal discriminator loss ${{\mathscr{L}}}_{{\rm{T}}}(\psi )$are minimized with respect to parameters *ϕ* and *ψ*, respectively; ReLU (*x*) = max(0,  *x*). The discriminator losses use a hinge loss formulation^26^:

4 $$\begin{array}{c}{{\mathscr{L}}}_{{\rm{D}}}(\varphi )={{\mathbb{E}}}_{{{\bf{X}}}_{1{\rm{:}}M+N},{\bf{Z}}}[{\rm{ReLU}}(1-{D}_{\varphi }({{\bf{X}}}_{M+1{\rm{:}}M+N}))+{\rm{ReLU}}(1+{D}_{\varphi }(G({\bf{Z}}{\rm{;}}{{\bf{X}}}_{1{\rm{:}}M})))].\end{array}$$

5 $$\begin{array}{c}{{\mathscr{L}}}_{{\rm{T}}}(\psi )={{\mathbb{E}}}_{{{\bf{X}}}_{1{\rm{:}}M+N},{\bf{Z}}}[{\rm{ReLU}}(1-{T}_{\psi }({{\bf{X}}}_{1{\rm{:}}M+N}))\,\,\,\,\,\,\,\\ \,\,\,\,\,\,\,\,\,\,\,\,\,\,+{\rm{ReLU}}(1+{T}_{\psi }({\rm{\{}}{{\bf{X}}}_{1{\rm{:}}M}{\rm{;}}G({\bf{Z}}{\rm{;}}{{\bf{X}}}_{1{\rm{:}}M}){\rm{\}}}))].\end{array}$$

#### Evaluation

During evaluation, the generator architecture is the same, but unless otherwise noted, full radar observations of size 1,536 × 1,280, and latent variables with height and width 1/32 of the radar observation size (48 × 40 × 8 of independent draws from a normal distribution), are used as inputs to the conditioning stack and latent conditioning stack, respectively. In particular, the latent conditioning stack allows for spatiotemporally consistent predictions for much larger regions than those on which the generator is trained.

For operational purposes and decision-making, the most important aspect of a probabilistic prediction is its resolution^36^. Specific applications will require different requirements on reliability that can often be addressed by post-processing and calibration. We develop one possible post-processing approach to improve the reliability of the generative nowcasts. At prediction time, the latent variables are samples from a Gaussian distribution with standard deviation 2 (rather than 1), relying on empirical insights on maintaining resolution while increasing sample diversity in generative models^24,37^. In addition, for each realization we apply a stochastic perturbation to the input radar by multiplying a single constant drawn from a unit-mean gamma distribution *G*(*α* = 5, *β* = 5) to the entire input radar field. Extended Data Figures 4 (UK) and 9 (US) shows how the post-processing improves the reliability diagram and rank histogram compared to the uncorrected approach.

#### Training

The model is trained for 5 × 10^5^ generator steps, with two discriminator steps per generator step. The learning rate for the generator is 5 × 10^−5^, and is 2 × 10^−4^ for the discriminator and uses Adam optimizer^38^ with *β*_1_ = 0.0 and *β*_2_ = 0.999. The scaling parameter for the grid cell regularization is set to *λ* = 20, as this produced the best continuous ranked probability score results on the validation set. We train on 16 tensor processing unit cores (https://cloud.google.com/tpu) for one week on random crops of the training dataset of size 256 × 256 measurements using a batch size of 16 per training step. The Supplementary Information contains additional comparisons showing the contributions of the different loss components to overall performance. We evaluated the speed of sampling by comparing speed on both CPU (10 core AMD EPYC) and GPU (NVIDIA V100) hardware. We generate ten samples and report the median time: for CPU the median time per sample was 25.7 s, and 1.3 s for the GPU.

### UNet baseline

We use a UNet encoder–decoder model as strong baseline similarly to how it was used in related studies^5,15^, but we make architectural and loss function changes that improve its performance at longer lead times and heavier precipitation. First, we replace all convolutional layers with residual blocks, as the latter provided a small but consistent improvement across all prediction thresholds. Second, rather than predicting only a single output and using autoregressive sampling during evaluation, the model predicts all frames in a single forward pass. This somewhat mitigates the excessive blurring found in ref. ^5^ and improves results on quantitative evaluation. Our architecture consists of six residual blocks, where each block doubles the number of channels of the latent representation followed by spatial down-sampling by a factor of two. The representation with the highest resolution has 32 channels which increases up to 1,024 channels.

Similar to ref. ^6^, we use a loss weighted by precipitation intensity. Rather than weighting by precipitation bins, however, we reweight the loss directly by the precipitation to improve results on thresholds outside of the bins specified by ref. ^6^. Additionally, we truncate the maximum weight to 24 mm h^−1^ as an error in reflectivity of observations leads to larger error in the precipitation values. We also found that including a mean squared error loss made predictions more sensitive to radar artefacts; as a result, the model is only trained with precipitation weighted mean average error loss.

The model is trained with batch size eight for 1 × 10^6^ steps, with learning rate 2 × 10^−4^ with weight decay, using the Adam optimizer with default exponential rates. We select a model using early stopping on the average area under the precision–recall curve on the validation set. The UNet baselines are trained with 4 frames of size 256 × 256 as context.

### Axial attention baseline

As a second strong deep learning-based baseline, we adapt the MetNet model^19^, which is a combination of a convolutional long short-term memory (LSTM) encoder^17^ and an axial attention decoder^39^, for radar-only nowcasting. MetNet was demonstrated to achieve strong results on short-term (up to 8 h) low precipitation forecasting using radar and satellite data of the continental USA, making per-grid-cell probabilistic predictions and factorizing spatial dependencies using alternating layers of axial attention.

We modified the axial attention encoder–decoder model to use radar observations only, as well as to cover the spatial and temporal extent of data in this study. We rescaled the targets of the model to improve its performance on forecasts of heavy precipitation events. After evaluation on both UK and US data, we observed that additional satellite or topographical data as well as the spatiotemporal embeddings did not provide statistically significant CSI improvement. An extended description of the model and its adaptations is provided in Supplementary Information section D.

The only prediction method described in ref. ^19^ is the per-grid-cell distributional mode, and this is considered the default method for comparison. To ensure the strongest baseline model, we also evaluated other prediction approaches. We assessed using independent samples from the per-grid-cell marginal distributions, but this was not better than using the mode when assessed quantitatively and qualitatively. We also combined the marginal distributions with a Gaussian process copula, in order to incorporate spatiotemporal correlation similar to the stochastically perturbed parametrization tendencies (SPPT) scheme of ref. ^40^. We used kernels and correlation scales chosen to minimize spatiotemporally pooled CRPS metrics. The best performing was the product of a Gaussian kernel with 25 km spatial correlation scale, and an AR(1) kernel with 60 min temporal correlation scale. Results, however, were not highly sensitive to these choices. All settings resulted in samples that were not physically plausible, due to the stationary and unconditional correlation structure. These samples were also not favoured by external experts. Hence, we use the mode prediction throughout.

### PySTEPS baseline

We use the PySTEPS implementation from ref. ^4^ using the default configuration available at https://github.com/pySTEPS/pysteps. Refs. ^3,4^ provide more details of this approach. In our evaluation, unlike other models evaluated that use inputs of size 256 × 256, PySTEPS is given the advantage of being fed inputs of size 512 × 512, which was found to improve its performance. PySTEPS includes post-processing using probability matching to recalibrate its predictions and these are used in all results.

### Performance evaluation

We evaluate our model and baselines using commonly used quantitative verification measures, as well as qualitatively using a cognitive assessment task with expert meteorologists. Unless otherwise noted, models are trained on years 2016–2018 and evaluated on 2019 (that is, a yearly split).

### Expert meteorologist study

The expert meteorologist study described is a two-phase protocol consisting of a ranked comparison task followed by a retrospective recall interview. The study was submitted for ethical assessment to an independent ethics committee and received favourable review. Key elements of the protocol involved consent forms that clearly explained the task and time commitment, clear messaging on the ability to withdraw from the study at any point, and that the study was not an assessment of the meteorologist’s skills and would not affect their employment and role in any way. Meteorologists were not paid for participation, since involvement in these types of studies is considered part of the broader role of the meteorologist. The study was anonymized, and only the study lead had access to the assignment of experimental IDs. The study was restricted to meteorologists in guidance-related roles, that is, meteorologists whose role is to interpret weather forecasts, synthesize forecasts and provide interpretations, warnings and watches. Fifty-six meteorologists agreed to participate in the study.

Phase 1 of the study, the rating assessment, involved each meteorologist receiving a unique form as part of their experimental evaluation. The axial attention mode prediction is used in the assessment, and this was selected as the most appropriate prediction during the pilot assessment of the protocol by the chief meteorologist. The phase 1 evaluation comprised an initial practice phase of three judgments for meteorologists to understand how to use the form and assign ratings, followed by an experimental phase that involved 20 trials that were different for every meteorologist, and a final case study phase in which all meteorologists rated the same three scenarios (the scenarios in Fig. 1a, and Extended Data Figs. 2 and 3); these three events were chosen by the chief meteorologist—who is independent of the research team and also did not take part in the study—as difficult events that would expose challenges for the nowcasting approaches we compare. Ten meteorologists participated in the subsequent retrospective recall interview. This interview involved an in-person interview in which experts were asked to explain the reasoning for their assigned rating and what aspects informed their decision-making. These interviews all used the same script for consistency, and these sessions were recorded with audio only. Once all the audio was transcribed, the recordings were deleted.

The 20 trials of the experimental phase were split into two parts, each containing ten trials. The first ten trials comprised medium rain events (rainfall greater than 5 mm h^−1^) and the second 10 trials comprised heavy rain events (rainfall greater than 10 mm h^−1^). 141 days from 2019 were chosen by the chief meteorologist as having medium-to-heavy precipitation events. From these dates, radar fields were chosen algorithmically according to the following procedure. First, we excluded from the crop selection procedure the 192 km that forms the image margins of each side of the radar field. Then, the crop over 256 km regions, containing the maximum fraction of grid cells above the given threshold, 5 or 10 mm h^−1^, was selected from the radar image. If there was no precipitation in the frame above the given threshold, the selected crop was the one with the maximum average intensity. We use predictions without post-processing in the study. Each meteorologist assessed a unique set of predictions, which allows us to average over the uncertainty in predictions and individual preference to show statistical effect.

Extended Data Figure 2 shows a high-intensity precipitation front with decay and Extended Data Fig. 3 shows a cyclonic circulation event (low-pressure area), both of which are difficult for current deep learning models to predict. These two cases were also assessed by all expert meteorologists as part of the evaluative study, and in both cases, meteorologists significantly preferred the generative approach (*n* = 56, *P* < 10^−4^) to competing methods. For the high-intensity precipitation front in Extended Data Fig. 2, meteorologists ranked first the generative approach in 73% of cases. Meteorologists reported that DGMR has “decent accuracy with both the shape and intensity of the feature … but loses most of the signal for embedded convection by *T* + 90”. PySTEPS is “too extensive with convective cells and lacks the organisation seen in the observations”, and the axial attention model as “highlighting the worst areas” but “looks wrong”.

For the cyclonic circulation in Extended Data Fig. 3, meteorologists ranked first the generative approach in 73% of cases. Meteorologists reported that it was difficult to judge this case between DGMR and PySTEPS. When making their judgment, they chose DGMR since it has “best fit and rates overall”. DGMR “captures the extent of precipitation overall [in the] area, though slightly overdoes rain coverage between bands”, whereas PySTEPS “looks less spatially accurate as time goes on”. The axial attention model “highlights the area of heaviest rain although its structure is unrealistic and too binary”. We provide additional quotes in Supplementary Information section C.6.

### Quantitative evaluation

We evaluate all models using established metrics^20^: CSI, CRPS, Pearson correlation coefficient, the relative economic value^22,41,42^, and radially averaged PSD. These are described in Supplementary Information section F.

To make evaluation computationally feasible, for all metrics except PSD, we evaluate the models on a subsampled test set, consisting of 512 × 512 crops drawn from the full radar images. We use an importance sampling scheme (described in Supplementary Information section A.1) to ensure that this subsampling does not unduly compromise the statistical efficiency of our estimators of the evaluation metrics. The subsampling reduces the size of the test set to 66,851 and Supplementary Information section C.3 shows that results obtained when evaluating CSI are not different when using the dataset with or without subsampling. All models except PySTEPS are given the centre 256 × 256 crop as input. PySTEPS is given the entire 512 × 512 crop as input as this improves its performance. The predictions are evaluated on the centre 64 × 64 grid cells, ensuring that models are not unfairly penalized by boundary effects. Our statistical significance tests use every other week of data in the test set (leaving *n* = 26 weeks) as independent units. We test the null hypothesis that performance metrics are equal for the two models, against the two-sided alternative, using a paired permutation test^43^ with 10^6^ permutations.

Extended Data Figure 4 shows additional probabilistic metrics that measure the calibration of the evaluated methods. This figure shows a comparison of the relative economic value of the probabilistic methods, showing DGMR providing the best value. We also show how the uncertainty captured by the ensemble increases as the number of samples used is increased from 1 to 20.

Extended Data Figure 5 compares the performance to that of an NWP, using the UKV deterministic forecast^44^, showing that NWPs are not competitive in this regime. See Supplementary Information section C.2 for further details of the NWP evaluation.

To verify other generalization characteristics of our approach—as an alternative to the yearly data split that uses training data of 2016–2018 and tests on 2019—we also use a weekly split: where the training, validation and test sets comprise Thursday through Monday, Tuesday, and Wednesday, respectively. The sizes of the training and test datasets are 1.48 million and 36,106, respectively. Extended Data Figure 6 shows the same competitive verification performance of DGMR in this generalization test.

To further assess the generalization of our method, we evaluate on a second dataset from the USA using the multi-radar multi-sensitivity (MRMS) dataset, which consists of radar composites for years 2017–2019^45^. We use two years for training and one year for testing, and even with this more limited data source, our model still shows competitive performance relative to the other baselines. Extended Data Figs. 7–9 compares all methods on all metrics we have described, showing both the generalization and skilful performance on this second dataset. The Supplementary Information contains additional comparisons on performance with different initializations and performance of different loss function components.

## Online content

Any methods, additional references, Nature Research reporting summaries, source data, extended data, supplementary information, acknowledgements, peer review information; details of author contributions and competing interests; and statements of data and code availability are available at 10.1038/s41586-021-03854-z.

## Supplementary information

Supplementary Information The Supplementary Information contains six sections: section A provides more details about the datasets used; section B gives more details of the generative model architecture; section C provides additional experiments mentioned in the methods; section D gives a more detailed description of the re-implemented baselines; section E provides context of the related work in nowcasting research; section F describes the precise definitions of the metrics used and their variants.

## Data Availability

Processed radar data for the UK yearly data split is released under a creative commons licence. A smaller dataset for exploratory analysis is freely available, and the full dataset (around 1 TB) is also available; for details, see github.com/deepmind/deepmind-research/tree/master/nowcasting. The associated datasets contain public sector information licensed by the Met Office under the UK Open Government Licence 3.0. For the raw data, other licences, and alternative time periods, the data from the UK can be obtained with appropriate agreements from the Met Office; see https://www.metoffice.gov.uk/research/weather/observations-research/radar-products or contact the Met Office Data Provisioning Team using dpt@metoffice.gov.uk. The multi-radar multi-sensor (MRMS) dataset is available with appropriate agreements from NOAA; see https://www.nssl.noaa.gov/projects/mrms/ or contact the MRMS data teams using mrms@noaa.gov. Source data are provided with this paper.

## Source data

Source Data Fig. 2
Source Data Fig. 3
Source Data Fig. 4

## Code availability

We rely on several open-source code frameworks including Iris (scitools-iris.readthedocs.io), Cartopy (scitools.org.uk/cartopy), TensorFlow (www.tensorflow.org), and Colab (colab.sandbox.google.com). We have also used open-source tools for PySTEPS (pysteps.github.io), and for Axial Attention (github.com/google-research/google-research/tree/master/axial). The pseudocode for the generative algorithm can be found in the file pseudocode.py in the Supplementary Information. All the neural architecture details and hyperparameters are described in Methods and Supplement. Alongside this model pseudocode, we have also released a pretrained version of the generative model available at github.com/deepmind/deepmind-research/tree/master/nowcasting.
