# Supplementary material for: Skilful precipitation nowcasting using deep generative models of radar
Source: Nature. 2021 Sep 29;597(7878):672–7. doi: 10.1038/s41586-021-03854-z (PMC8481123; doi:10.1038/s41586-021-03854-z)
Supplement: Supplementary file 1 — The Supplementary Information contains six sections: section A provides more details about the datasets used; section B gives more details of the generative model architecture; section C provides additional experiments mentioned in the methods; section D gives a more detailed description of the re-implemented baselines; section E provides context of the related work in nowcasting research; section F describes the precise definitions of the metrics used and their variants. [file 41586_2021_3854_MOESM1_ESM.pdf]

nature portfolio

https://doi.org/10.1038/s41586-021-03854-z

Supplementary information

Skilful precipitation nowcasting using deep generative models of radar

In the format provided by the
authors and unedited

Supplementary Materials
Skillful Precipitation Nowcasting using Deep Generative Models of Radar

Suman Ravuri, Karel Lenc, Matthew Willson, Dmitry Kangin, Remi Lam, Piotr Mirowski, Megan Fitzsimons, Maria Athanassiadou, Sheleem Kashem, Sam Madge, Rachel Prudden, Amol Mandhane, Aidan Clark, Andrew Brock, Karen Simonyan, Raia Hadsell , Niall Robinson, Ellen Clancy , Alberto Arribas, and Shakir Mohamed

This material contains six sections: Section A provides more details about the datasets used; Section B gives more details of the generative model architecture; Section C provides additional experiments mentioned in the methods; Section D gives a more detailed description of the re-implemented baselines; Section E provides context of the related work in nowcasting research; Section F describes the precise definitions of the metrics used and their variants.

A. Additional Dataset Details

Here, we provide additional information regarding datasets. We detail the importance sampling scheme we employed to favor heavy rainfall examples in the dataset (Supplement A.1). We then provide a description of the data used to construct the US datasets (Supplement A.2). Finally, we provide the precipitation rate statistics of our datasets (Supplement A.3).

A.1. Importance Sampling Scheme

In the Methods we described the use of an importance sampling scheme to increase the frequency with which crops with rain were encountered during training. Most regions in the radar composite contain little or no rainfall, and such regions typically contribute little to the variance of our estimators for evaluation metrics and loss gradients. We can take advantage of this to reduce the computational cost of evaluations without unduly compromising the statistical efficiency of these estimators. Specifically, we create sub-sampled datasets using an importance sampling scheme [1] that favors heavier-rainfall examples, and use importance-weighted sums to address the bias which this introduces.

Starting from a full-frame example of size $T \times H \times W$ —for time-length T, height H and width W—the scheme samples smaller examples (i.e., crops) of size $T \times h \times w$, including examples of heavier rainfall with higher probability. Given one of these smaller examples x_n, the probability of sampling x_n is a function of its rain rates $x_{n,c}$, where c indexes over all $C = T \times h \times w$ grid cells in the example. Saturated values are computed from rain rates as $x_{n,c}^{\text{sat}} = 1 - \exp(-x_{n,c}/s)$, where s is a saturation constant and $x_{n,c}$ is set to zero for masked grid cells. These are averaged, scaled and clipped to give an acceptance probability

$$q_n = \min\{1, q_{\min} + \frac{m}{C} \sum_c x_{n,c}^{\text{sat}}\}, \tag{6}$$

where $q_{\min}$ is the minimum probability of inclusion and m is a multiplier controlling the overall inclusion rate. See Supplementary Table 1 for the values used for each dataset.

We consider different crops when sampling for the training, validation or test sets. For validation and test, we only consider crops whose vertical and horizontal offsets are multiples of 64. The probability of a given crop x_n being included in the dataset is thus q_n as detailed above. In particular, for the 512×512 test sets, for which we only compute metrics on the central 64×64 grid cells, this ensures that every grid cell has a chance of being included in the evaluation, while reducing the overlap of crops. For training, we want to create datasets as large as possible. So, to ensure that examples can be sampled at all possible offsets, we first consider all the crops with a vertical and horizontal offsets multiple of 32. If one of those examples x_n is accepted with probability q_n, instead of including it in the dataset, we add uniform

Supplementary Table 1 | **Summary of parameters used in generating full frame and sub-sampled data sets.**

Dataset	Training Sub-sampled		Validation Sub-sampled		Test Sub-sampled		Test Full-frame	
	UK	US	UK	US	UK	US	UK	US
s	1.0	1.0	1.0	1.0	10.0	30.0	-	-
m	0.1	0.1	2.2	0.2	1.0	0.2	-	-
$q_{\min}$	2×10^{-4}	2×10^{-4}	5×10^{-3}	5×10^{-3}	2×10^{-5}	2.5×10^{-4}	-	-
T	24	20	24	20	24	20	24	20
h	256	256	256	256	512	512	1536	3584
w	256	256	256	256	512	512	1280	7168
Spatial offset	32	32	256	256	64	64	-	-
Random offset	True	True	False	False	False	False	False	False
Temporal offset between examples (min)	5	6	20	24	20	24	20	24

random offsets between 0 and 32 grid cells horizontally and vertically, defining a new example $x_{n'}$. The example $x_{n'}$ is then added to the dataset and its inclusion probability is effectively $q'_{n'} = q_n/32^2$.

When computing evaluation metrics on the subsampled validation and test sets, we use unbiased importance-weighted estimators in place of sums over the full dataset of examples $\{x_1, \ldots, x_N\}$. We estimate $\sum_{n=1}^{N} S(x_n)$ using the subsample $\{x_{n_1}, \ldots, x_{n_L}\}$ as $\sum_{l=1}^{L} q_{n_l}^{-1} S(x_{n_l})$.

We also explored using importance sampling weights at training time to correct bias in our estimates of loss gradients. We found no significant advantage from this and therefore we did not use importance weights at training time.

A.2. United States Dataset

To train and evaluate models of precipitation nowcasting over the United States, we use radar composites from the Multi-Radar Multi-Sensor (MRMS) system [2, 3]. The data is acquired with a network of 146 WSR-88D radars covering the conterminous US and 30 Canadian radars[2]. We refer to [2] for details on how reflectivity fields are converted to precipitation rates and how precipitation classification informs this transformation. The radar composites cover latitudes between 20° and 55° North and longitudes between 130° and 60° West. The resolution of the 3584 × 7168 composites is of 0.01° in both latitude and longitude directions; this is equivalent to 1.11 km uniformly in the North-South direction. However, in the West–East direction 0.01° represents about 0.6 km at the top of the image and about 1 km at the bottom. Similar to the UK data, missing values are identified by a negative value, which is used to mask irrelevant grid cells during training and evaluation. To construct our datasets, we use the radar composites collected every 2 minutes between January 1, 2017 and December 31, 2019. When generating an example from a sequence of radar composites, we downsample the temporal resolution of the data by 3×, ignoring 2 composites out of 3, effectively making predictions by increments of 6 minutes. We perform this downsampling operation since, while the nominal temporal resolution is two minutes, the duration to complete a volume scan by the radars varies from 3 to 10 minutes. As a result, precipitation dynamics present “skipping" patterns, with measurements from different radars updating asynchronously (see [2]). Reducing the effective temporal resolution to 6 minutes mitigates this “skipping" effect and makes the temporal resolution comparable to the UK dataset. We cap the rain rates at the value of 1024 mm/hr.

[2] https://www.roc.noaa.gov/WSR88D/Maps.aspx

Supplementary Table 2 | **Rainfall Distributions (in percentage).** Shown for the UK and US yearly test set per location and dataset type. Statistics are computed across 15 consecutive frames from 10^4 randomly drawn examples for the sub-sampled datasets, and 10^3 examples for the full frame datasets.

Dataset<br>Interval in mm/hr	UK<br>Full-Frame	UK<br>Subs.	US<br>Full-Frame	US<br>Subs.
$= 0.0$	89.18	69.14	94.52	79.9
$(0, 0.1]$	1.72	3.61	0.00	0.00
$(0.1 - 1.0]$	5.96	16.18	3.46	9.82
$(1.0 - 4.0]$	2.75	9.59	1.66	7.97
$(4.0 - 5.0]$	0.16	0.62	0.11	0.74
$(5.0 - 8.0]$	0.16	0.64	0.13	0.91
$(8.0 - 10.0]$	0.03	0.11	0.03	0.21
> 10.0	0.03	0.11	0.08	0.45

A.3. Dataset Statistics

In Supplementary Table 2, we show summary statistics of the distribution of the rainfall amount in mm/hr for both the UK and US dataset. These statistics show the high proportion of no rain grid cells, differences in high-intensity grid cells between the US and UK data, and the effect of the importance sampling scheme towards higher intensity grid cells to support learning.

B. DGMR Architectural Details

The generator consists of two main modules: the conditioning stack, which creates a conditioning representation from previous radar observations; and the sampler, which generates 18 predictions of future radar from the conditioning representation. These descriptions accompany the schematic description in Extended Data Figure 1.

The *conditioning stack* is a feed-forward convolutional neural network (Extended Data Figure 1a) that generates the conditioning representation from four radar observations. First, each $256 \times 256 \times 1$ radar observation is converted to a $128 \times 128 \times 4$ input by stacking 2×2 patches into the channel layer (space-to-depth). Then, each radar observation is processed separately to ensure that each frame is processed in the same way since they are all the same data. We use four downsampling residual blocks (D Block in Extended Data Figure 1b), which decreases the resolution and increases the number of channels by a factor of two. The four outputs of each residual block are concatenated across the channel dimension, and, for each output, a 3×3 spectrally normalized convolution is applied to reduce the number of channels by a factor of two, followed by a rectified linear unit. This yields a stack of conditioning representations of sizes $64 \times 64 \times 48$, $32 \times 32 \times 96$, $16 \times 16 \times 192$, and $8 \times 8 \times 384$.

The *sampler* (Extended Data Figure 1a), which is a stack of four ConvGRU units, uses the conditioning representations as initial states for each of its recurrent modules. Along with the initial states, 18 copies (one for each lead time) of an $8 \times 8 \times 768$ latent representation are given as input to the lowest-resolution ConvGRU block. This representation is generated by the latent conditioning stack, a small feed-forward convolutional network that converts an $8 \times 8 \times 8$ input to the latent representation by gradually increasing the number of channels.

For the *latent conditioning stack*, entries in the $8 \times 8 \times 8$ input are independent draws from a normal distribution $\mathcal{N}(0, 1)$. The first two dimensions of the input are height and width, which are 1/32 of the height and width of the $256 \times 256 \times 1$ radar observations. The latent conditioning stack comprises one

3×3 convolution, three L Blocks, a spatial attention module [4, 5], and one L Block. The L Block is a modified residual block designed specifically for increasing the number of channels of its respective input.

As described, the output of the latent conditioning stack is repeated 18 times and is used as input to the lowest resolution ConvGRU. The output of each ConvGRU is then upsampled to an input of the next ConvGRU with one spectrally normalized convolution and two residual blocks that process all 18 temporal representations independently. The second residual block doubles the input's spatial resolution with nearest neighbor interpolation, and halves its number of channels. After the last ConvGRU, the intermediate feature vector is of size $128 \times 128 \times 48$. After batch normalization, a ReLU and a 1×1 spectrally normalized convolution is applied, yielding an output of size $128 \times 128 \times 4$. Similar to super-resolution, this is converted to 18 predictions of size $256 \times 256 \times 1$ with a depth-to-space operation.

The spatial and temporal discriminators used to train DGMR are similar to [6], and either operate on predictions (for generator steps), or predictions and targets (for discriminator steps). The *spatial discriminator* picks uniformly at random 8 out of 18 lead times, which are first downsampled to $128 \times 128 \times 1$ using 2×2 mean pooling, and then converted to a $64 \times 64 \times 4$ input by stacking 2×2 patches into the channel layer (space-to-depth). This is followed by five residual blocks (D Block), each of which halve the resolution while doubling the number of channels. The first D Block does not apply a ReLU before the first 3×3 convolution. The outputs of the five blocks are $32 \times 32 \times 48$, $16 \times 16 \times 96$, $8 \times 8 \times 192$, $4 \times 4 \times 384$, and $2 \times 2 \times 768$, respectively. After being processed by one more D Block that preserves the spatial resolution and number of channels, the representations are sum-pooled along the height and width dimensions. The 8 resulting representations are inputs to a spectrally normalized linear layer, which are then summed together before a ReLU is applied for binary classification output.

The input to the *temporal discriminator* is a sequence comprised of the four contextual radar frames concatenated along the time axis to either the predictions (for generator steps), or the predictions or targets (for discriminator steps). A random crop of height and width 128×128 is extracted from the sequence, and each frame in the sequence is then converted to $64 \times 64 \times 4$ using a space-to-depth operation. This output is processed by two 3D Blocks, which mimic the processing of the first two D Blocks in the spatial discriminator, but with $3 \times 3 \times 3$ spectrally normalized convolutions. The first 3D Block does not apply a ReLU before the first $3 \times 3 \times 3$ convolution. Each frame of the resulting length-five $16 \times 16 \times 96$ representation is processed by four residual blocks with same architecture as that of the spatial discriminator. The remaining steps are identical to those after the last D Block of the spatial discriminator.

C. Additional Experimental Analysis

C.1. Additional Quantitative Evaluation on Yearly Data Splits

We provide additional quantitative evaluation for the models trained on the yearly train-validation-test data split.

Training variability. In order to quantify the variance of the training algorithm, we trained five instances of DGMR, each trained with a different sequence of training examples. We show the performance for CSI and pooled CRPS (with one standard deviation error bars) in Supplementary Figures, 1 and 2, respectively.

Justifying the choice of loss. In Supplementary Figure 3, we show the influence of the different combinations of losses on the test set performance. It can be seen that the proposed combination of losses

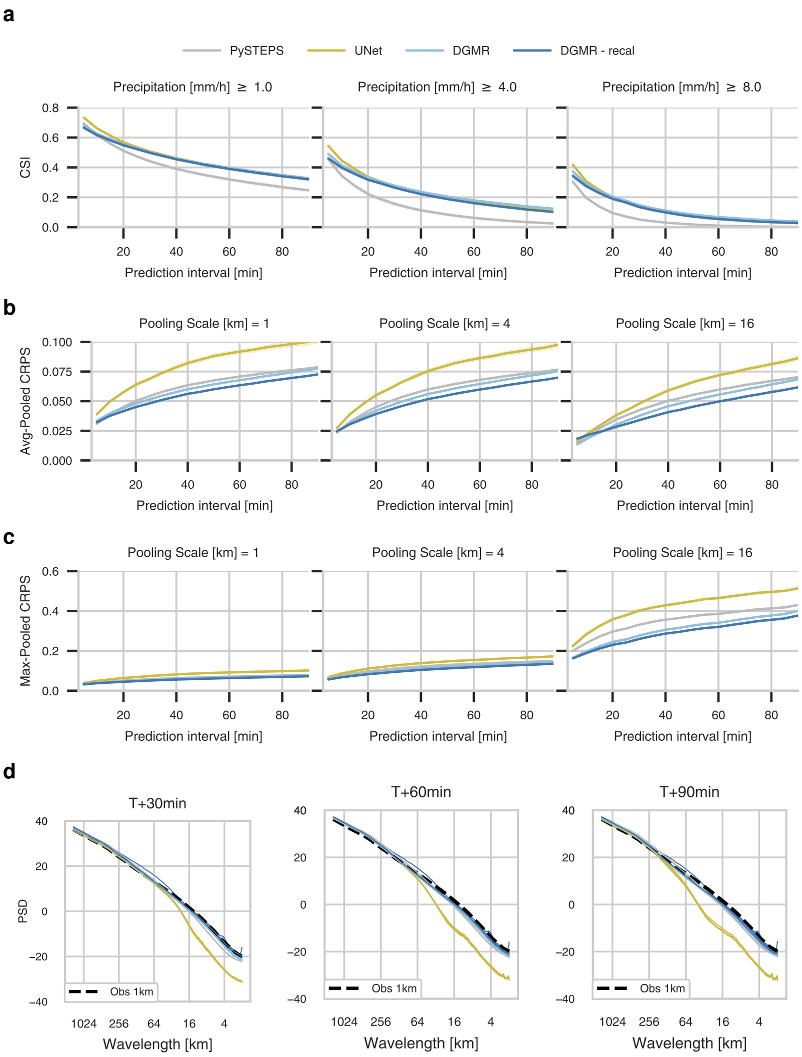

Supplementary Figure 1 | **Verification scores for the United Kingdom in 2019 for five DGMR and UNet initializations and five runs of PySTEPS. a:** Critical Success Index across 20 samples of different models across precipitation thresholds 1 mm/hr (left), 4 mm/hr, 8 mm/hr (right) with 95% confidence interval. Each UNet initialization generates a single deterministic prediction. **b:** Average-pooled CRPS of various models for original predictions (left), average rain rate over a 4 km × 4 km catchment area (middle), and average rain rate over a 16 km × 16 km catchment area (right) with 95% confidence interval. **c:** Max-pooled CRPS of various models for original predictions (left), maximum rain rate over a 4 km × 4 km catchment area (middle), and maximum rain rate over a 16 km × 16 km catchment area (right) with 95% confidence interval. **d:** Radially-averaged power spectral density for full-frame 2019 predictions for different models across the 5 initializations.

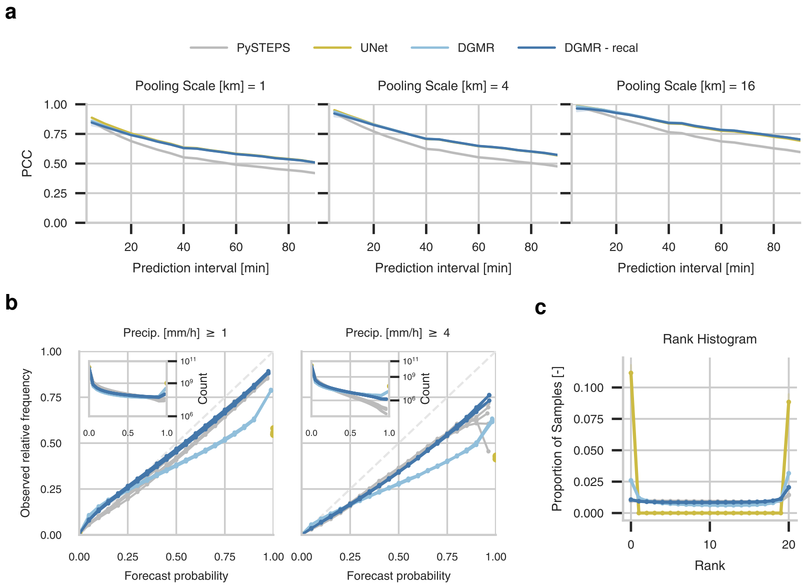

Supplementary Figure 2 | **Further verification scores for the United Kingdom in 2019 for five initializations. a:** Pearson Correlation of various models for original predictions (left), average rain rate over a 4 km × 4 km catchment area (middle), and average rain rate over a 16 km × 16 km catchment area (right) with 95% confidence interval. **b:** Reliability Plot across individual initializations. **c:** Rank Histogram with 95% confidence interval.

is important for obtaining favorable results across a combination of metrics. For example, using only the per-grid-cell regularization loss leads to best results with regards to per-grid-cell CSI (a), however it leads to a significant increase of the CRPS metrics (c) and unfavorable spatial frequency (PSD) characteristics (d).

Evaluation by Precipitation Type Supplementary Figure 4 compares the performances of competing methods against different rain types. We annotated examples covering the entire United Kingdom during 2019 as belonging to several rain types. These examples were annotated by an author of the paper but not involved in model development. These annotations were then reviewed and approved by an independent forecaster at the Met Office. These precipitation-event types are: frontal, non-frontal, or mixed type precipitation; precipitation with coherent motion, incoherent motion, or mixed type; and scattered, non-scattered, or mixed scattered and non-scattered precipitation. In nearly all cases, DGMR outperformed competing methods on both CRPS and CSI metrics. DGMR performs particularly well for non-frontal rain (panels a,b), which is an important result as non-frontal rain is known to be very difficult to predict [7].

To assign labels for rain types, each example is labeled with the following properties. An example is labeled as frontal if directed movement of air masses with a visible front line exists, non-frontal if no precipitation exists, or mixed if part of the example contains a front. An example is labeled scattered if scattered rain exists, unscattered if none exists, and mixed if this changes during the example. Finally, the motion of precipitation is labeled coherent if the precipitation field was moving coherently, in the same direction, non-coherent otherwise, or mixed if both types exist in an example. This last property is used to classify whether or not precipitation is advective. We then compared the performance of our model and baselines using the quantitative metrics across the different subtypes.

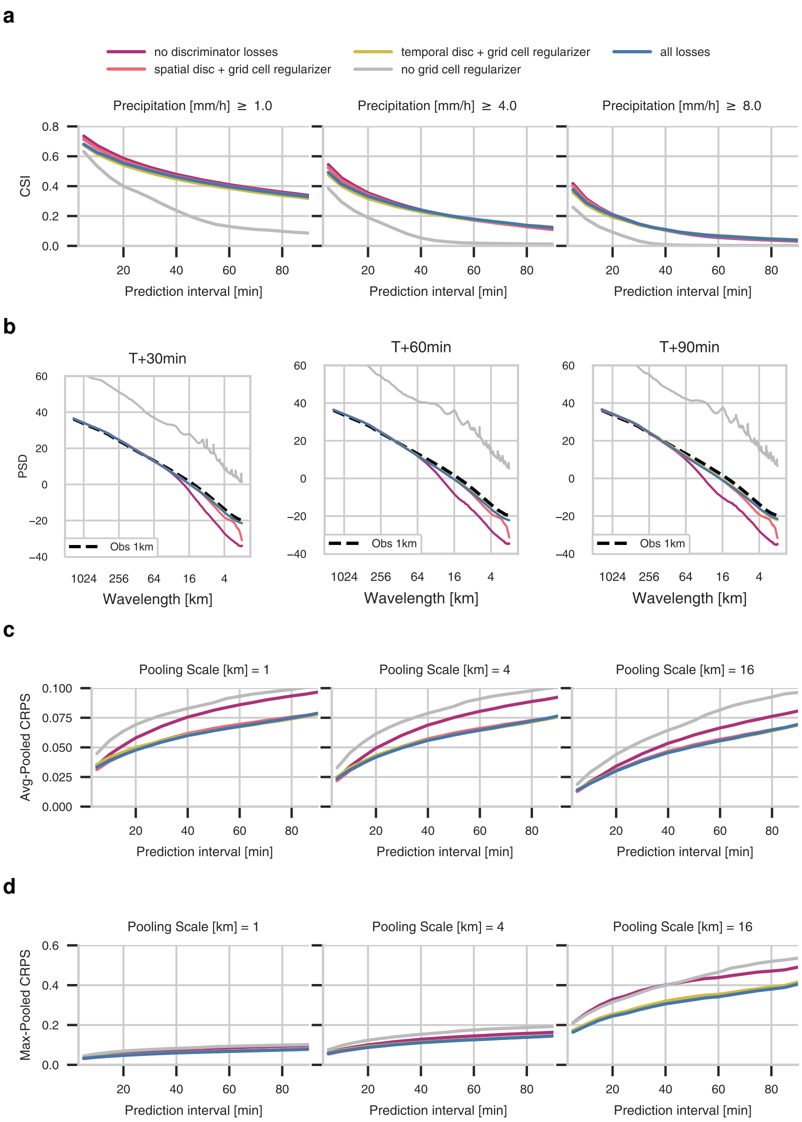

Supplementary Figure 3 | **Verification scores for the United Kingdom in 2019 with ablations of DGMR.** Compared losses are grid cell regularization with no discriminator losses, spatial discriminator with grid cell regularization, temporal discriminator with grid cell regularization, discriminator losses without grid cell regularization, and all three losses (the two discriminator losses and the regularization). **a:** Critical Success Index across 20 samples of different models across precipitation thresholds 1 mm/hr (left), 4 mm/hr, 8 mm/hr (right). **b:** Radially-averaged power spectral density for full-frame 2019 predictions for different models. **c:** CRPS of various models for original predictions (left), average rain rate over a 4 km × 4 km catchment area (middle), and average rain rate over a 16 km × 16 km catchment area (right). **c:** CRPS of various models for original predictions (left), maximum rain rate over a 4 km × 4 km catchment area (middle), and maximum rain rate over a 16 km × 16 km catchment area (right).

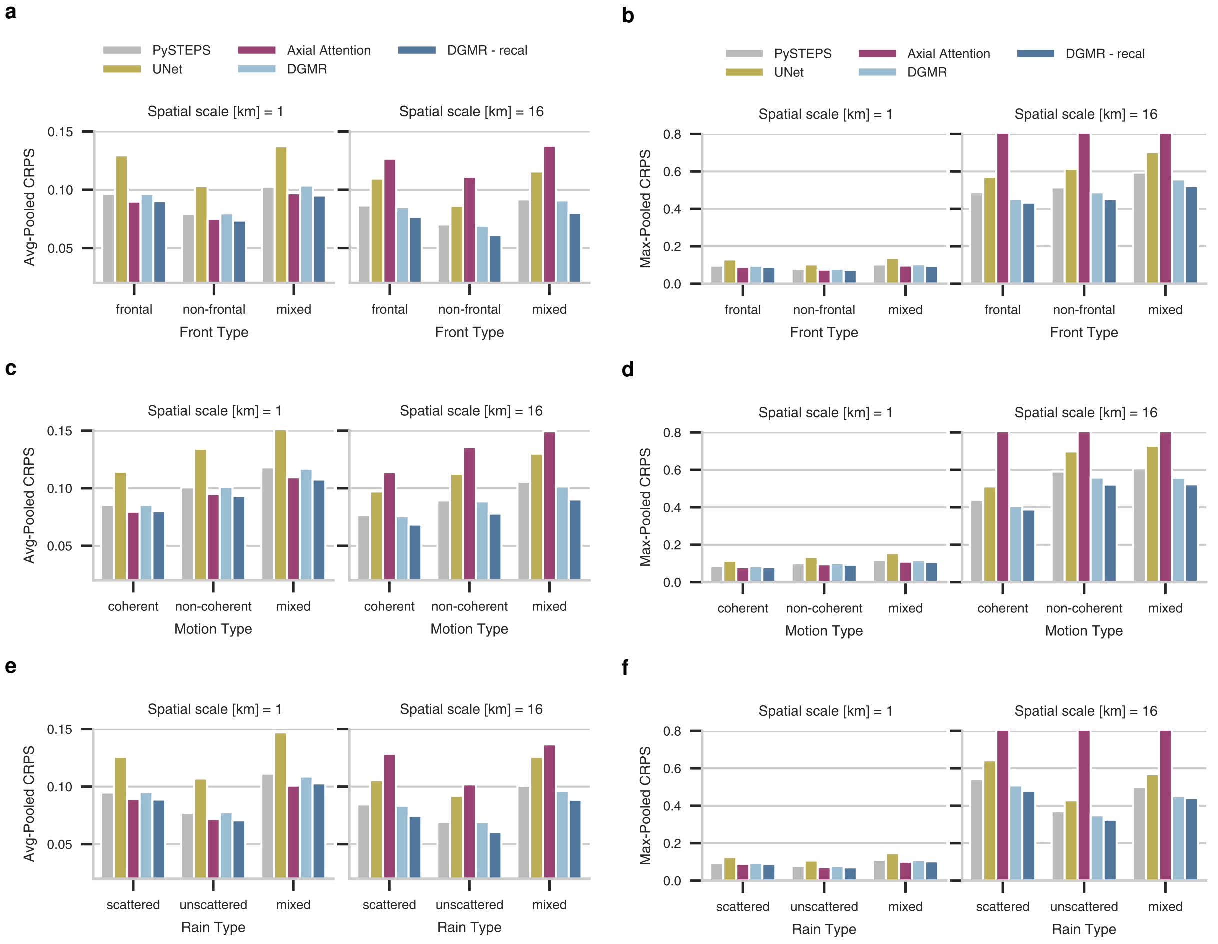

Supplementary Figure 4 | **Rain-type analysis for predictions T+90 min lead time, comparing CRPS metrics at 1 km and 16 km spatial scales for the United Kingdom. a:** Average pooled Continuous Ranked Probability Score across 20 samples of different models at scales 1 km and 16 km, for frontal, non-frontal, and mixed-type precipitation events. UNet generates a single deterministic prediction. **b:** Max-pooled CRPS across 20 samples of different models for frontal, non-frontal, and mixed-type precipitation events. **c:** Avg-pooled CRPS for coherent, non-coherent, and mixed-type precipitation events. **d:** Max-pooled CRPS for coherent, non-coherent, and mixed-type precipitation events. **e:** Avg-pooled CRPS for scattered, non-scattered, and mixed-type precipitation events. **f:** Max-pooled for scattered, non-scattered, and mixed-type precipitation events.

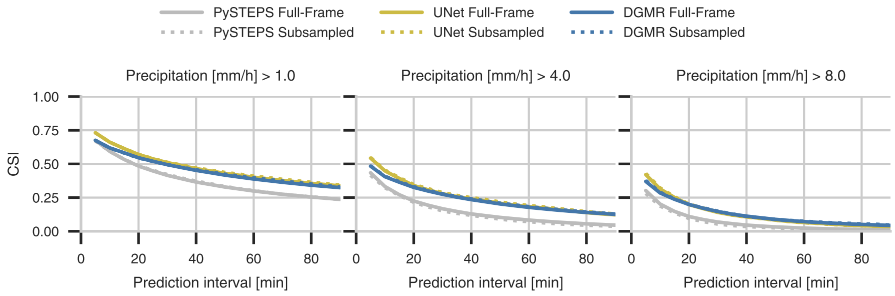

Supplementary Figure 5 | **Verification scores for the United Kingdom on yearly splits show no significant difference between the sub-sampled and full-frame datasets.** Results computed over an ensemble of 5 samples for PySTEPS and DGMR. Datasets are specified in table 1.

C.2. NWP Results

The Methods and Extended Data 5 reference comparison to how the NWP would perform within the nowcasting timescales. We perform a basic comparison against the NWP performance. We use the rainflux variable from the Met Office Deterministic UK model (UKV) [8]. The rainflux variable in this model has 5 minutes temporal and 1.5 km spatial resolution, which we upsample to the OSGB36 1 km scale reference grid. In practice, data assimilation is performed multiple times a day to initialize a new NWP with the most recent observations. Therefore, we conduct the evaluation of the NWP baseline by restricting the test set to examples that align the first prediction target with the initialization of a NWP. This advantages the NWP in two ways. First, it evaluates its performance in the ideal case where it has just been updated with observations. Second, the NWP is given instantaneous access to its prediction after initialization. In an operational setting, the time taken by data assimilation would make the first few predictions of the model unavailable in real time. We use data generated by NWPs initialized four times a day to construct this evaluation over the UK. For the CSI and CRPS, we use the 512×512 test set containing 3,704 examples. For PSD, we use the 1536×1280 test set containing 1,409 examples. Note that because of their reduced size, these datasets contain a limited number of heavy precipitation events. Supplementary Figure 5 shows a comparison of PySTEPS, UNet, and DGMR to NWP on CSI, CRPS and PSD. Overall, the NWP performs poorly compared to other baselines and DGMR at nowcasting timescales on CSI and CRPS, and since it preserves physical properties, makes predictions with good spectral characteristics.

C.3. Empirical Comparison of the Sub-sampled and Full-frame Dataset

We provide additional details of the empirical comparison of the 512×512 sub-sampled and 1536×1280 full-frame datasets here. In Supplementary Figure 5, we show a quantitative comparison of CSI scores obtained on the full-frame UK dataset (yearly splits, test set) versus the results obtained on the sub-sampled dataset. Since computing the predictions for the full-frame dataset is computationally prohibitive, we evaluate the STEPS and DGMR only on an ensemble of 5 samples (instead of 20, which is used in other experiments). We observe no quantitative difference, further motivating the use of the 512×512 sub-sampled dataset for all metrics but PSD.

C.4. Computational Speed

In Supplementary table 3, we show execution speed of some of the selected models. We evaluate the speed of sampling by comparing speed on both CPU (10 cores of AMD EPYC processor) and GPU (NVIDIA V100) hardware for the deep learning models. We generate 10 samples and report the median time. As

Supplementary Table 3 | **Execution speed of selected models.** Computed for a single samples of the full-resolution UK data sample (1536 × 1280 locations), and reported as median time across 10 samples.

Model	PySTEPS	UNet	Generative Method
CPU Speed [s]	69.54	2.78	25.66
GPU Speed [s]	-	0.1	1.27

the Axial Attention model requires tiling at evaluation, it is not directly comparable and is not included here.

C.5. Additional Visualizations

For reference we include two additional types of visualizations here: predictions over the full UK, and postage stamp plots to visualize the ensemble variability. Figure 6 shows full frame predictions of DGMR for case studies in the main paper and extended data. Figure 7 shows the same full UK prediction but with the recalibration for DGMR.

Figures 8,9,10 show postage stamp plots with 6 different realisations from the ensemble to provide insight into the ensemble variability.

C.6. Additional Forecaster Responses

To further support the statement that forecasters made deliberate judgements of the predictions by relying on their expertise, rather than being swayed by realistic looking images, we list additional quotes from the phase 2 restrospective recall interviews. Emphasis below is our own.

F18 "... between A and C I looked for where it had the moderate bright bursts, *because that's what you look for when you want to see which model is doing better* when you're verifying forecasting. At T+60 for example A doesn't have any of the moderate bursts whereas C does." [A=STEPS ; C=Proposed]

F9 "I think B does a good representation at T+30, *I don't think you can expect a model to do much more than that*. Because it's got all the light stuff, it's got the heavier bursts in the south and the north and as you continue through it's still reasonable good even at T+90. It doesn't have the heavier stuff in far North but the gaps aren't as good. The only stuff it doesn't pick up is towards the edge of the radar, but as it's towards the edge of the radar with it being that light I wonder how realistic that representation is. I wonder if the models are more right than the radar here." [A=Axial Attention; B: Proposed; C=STEPS]

F9 "The reason I chose C was *I like the the way it was picking up the distribution, particularly over the Valley* area, and then also looking slightly further towards the South are kind of where it was picking up with the heavier stuff down there. " [A=Proposed; B=STEPS; C=Axial Attention]

F85 "I *like how A does not attempt to look realistic*, but appears to give the best spatial sense of the ppn, and highlight broad zones where heavier ppn is possible. B is reasonable but not as good spatially as A, and C is by far the worst." [A=Axial Attention; B=Proposed; C=STEPS]

F87 "*Radar likely overestimating rates*, so lower rainfall rates preferred."

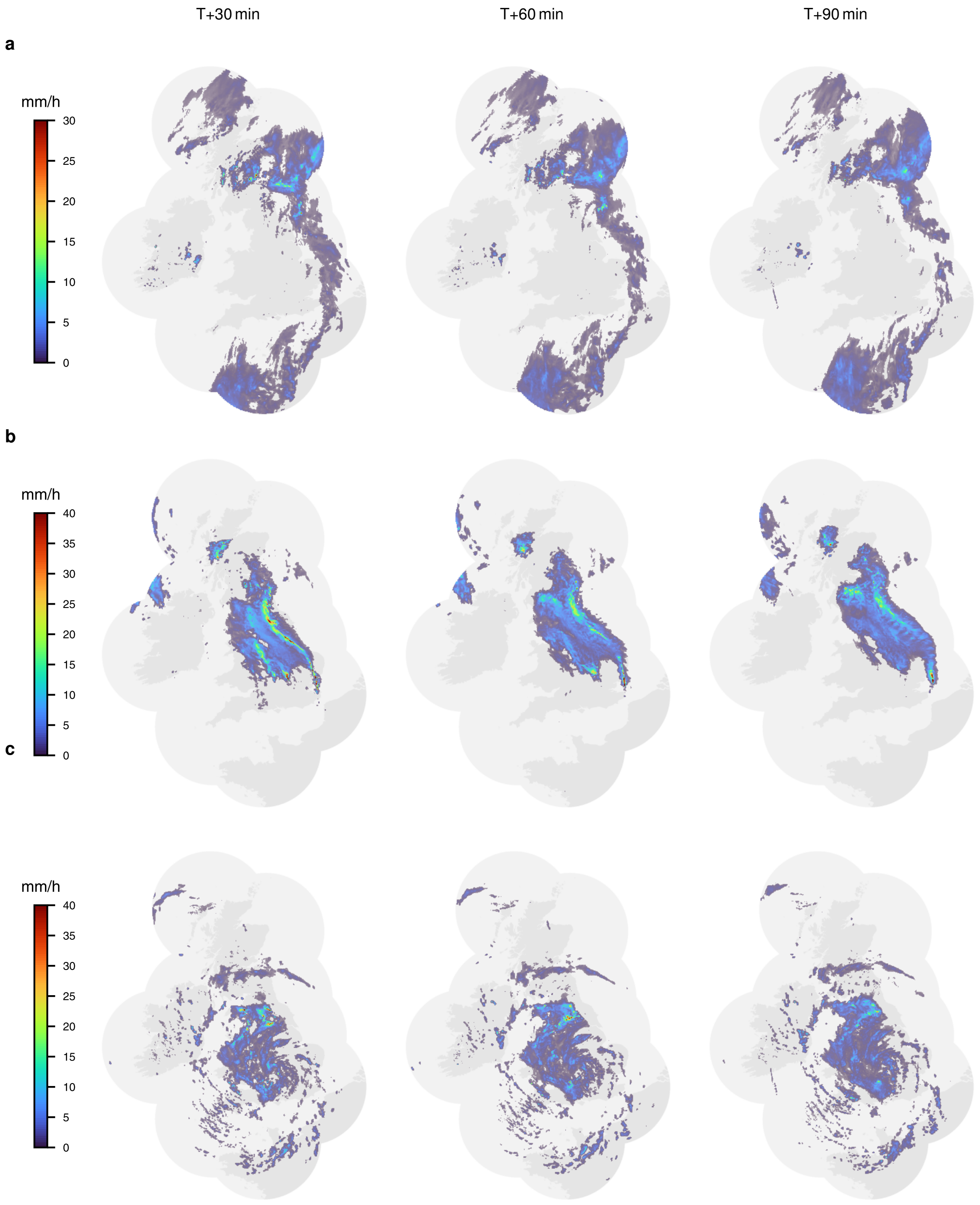

Supplementary Figure 6 | **Full frame predictions of DGMR for case studies in the paper.**
a: Precipitation event starting at T=2019-06-24 at 16:15 UK, showing convective cells over eastern Scotland. **b:** Precipitation event starting at 2019-07-24 at 03:15 UK, showing two separate banded structures of intense rainfall in the north-east and south-west over northern England. **c:** Precipitation event starting 2019-07-30 at 15:15 UK, showing a pattern of precipitation around a low pressure area which is slow moving, resulting in the cyclonic banded structures over England. Maps produced with Cartopy and SRTM elevation data [9].

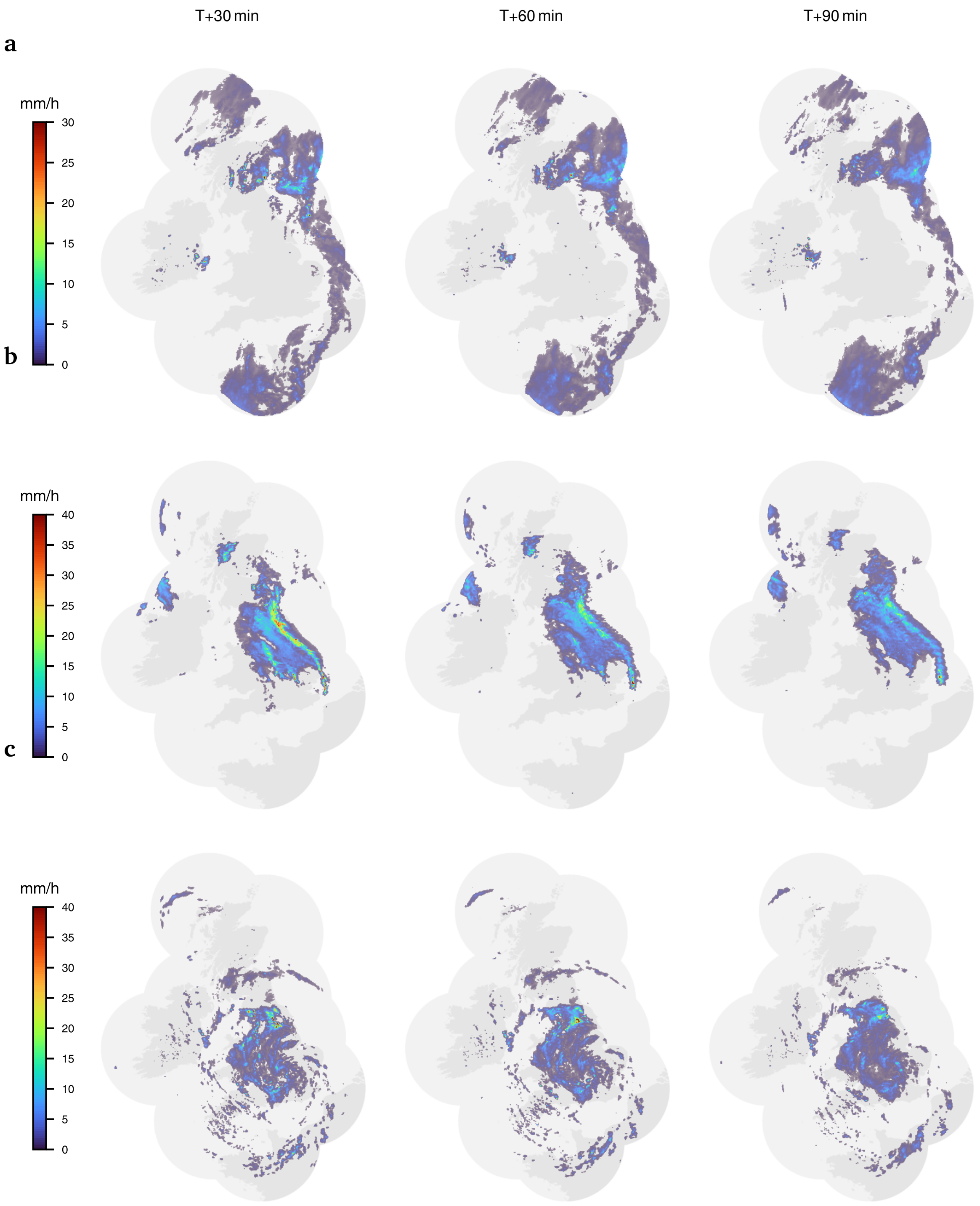

Supplementary Figure 7 | **Full frame predictions of DGMR after calibration for case studies in the paper.**
a: Precipitation event starting at T=2019-06-24 at 16:15 UK, showing convective cells over eastern Scotland. **b:** Precipitation event starting at 2019-07-24 at 03:15 UK, showing two separate banded structures of intense rainfall in the north-east and south-west over northern England. **c:** Precipitation event starting 2019-07-30 at 15:15 UK, showing a pattern of precipitation around a low pressure area which is slow moving, resulting in the cyclonic banded structures over England. Maps produced with Cartopy and SRTM elevation data[9].

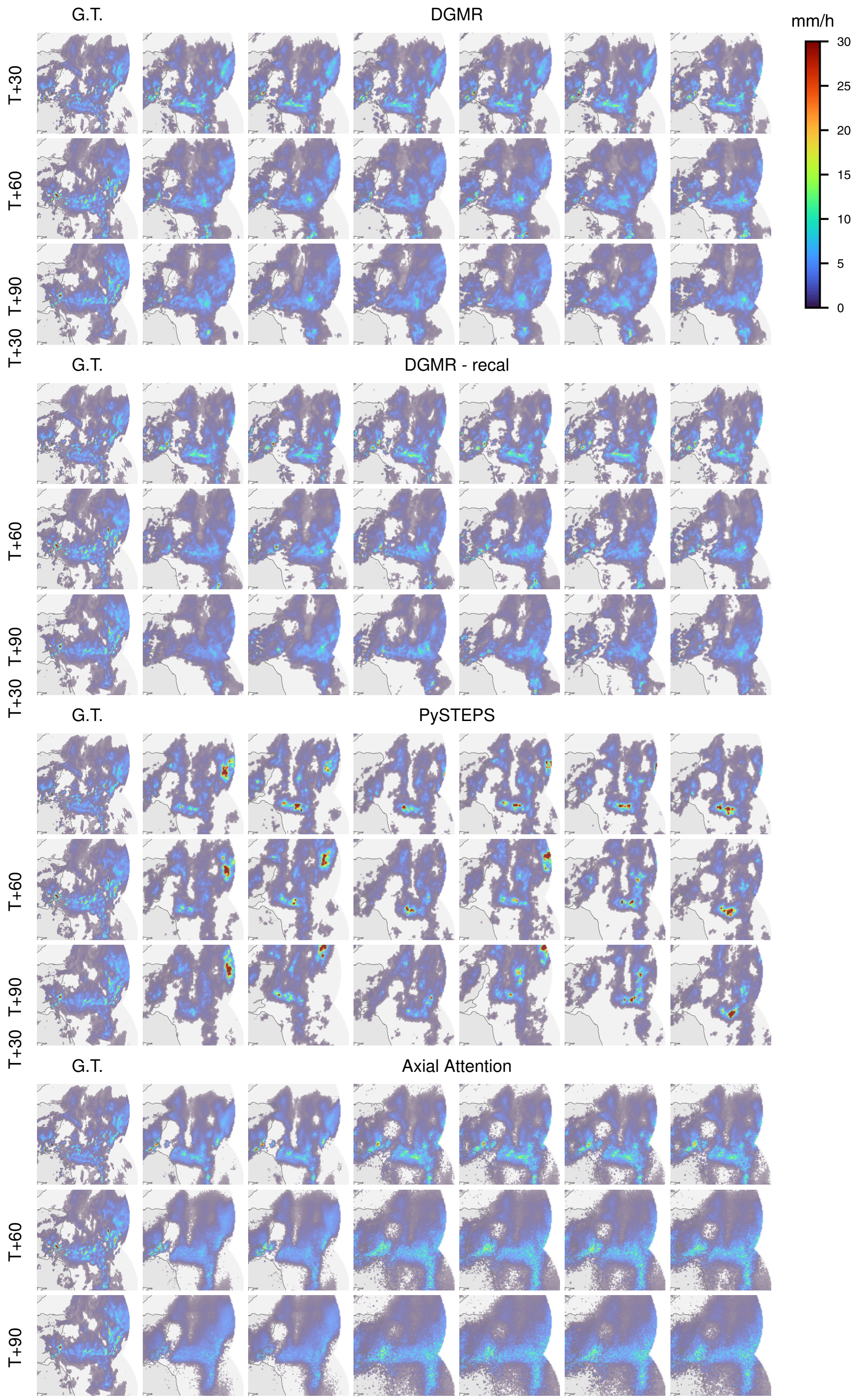

Supplementary Figure 8 | **Postage stamp plots.** Ground Truth (left) and 6 samples. The rows for each method show predictions for T+30 , T+60 , and T+90 mins lead time, for a challenging precipitation event showing convective cells over eastern Scotland. Images are 256 km × 256 km. Maps produced with Cartopy and SRTM elevation data[9].

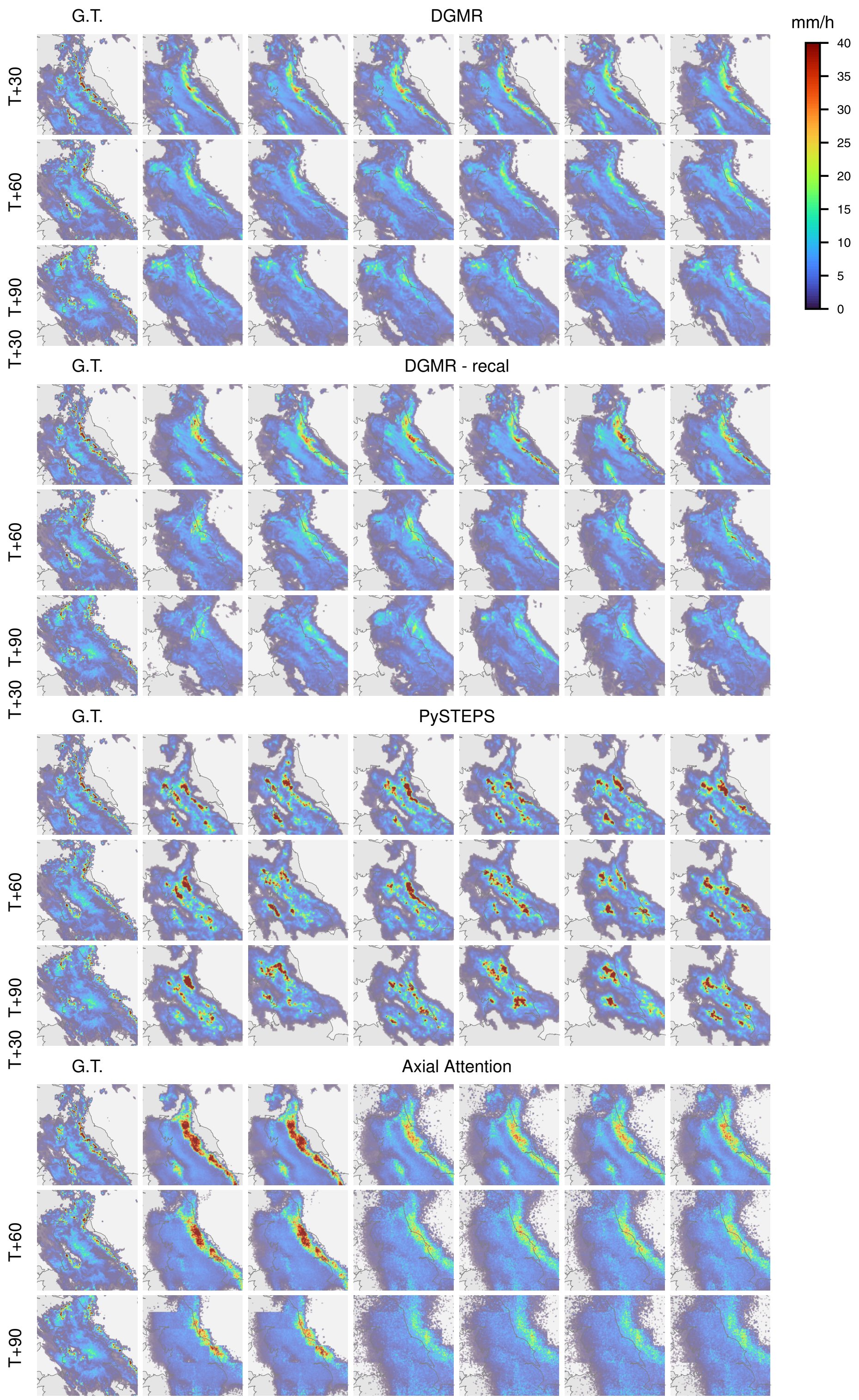

Supplementary Figure 9 | **Postage stamp plots**. Ground Truth (left) and 6 samples. The rows for each method show predictions for T+30 , T+60 , and T+90 mins lead time, for a challenging precipitation event showing two separate banded structures of intense rainfall in the north-east and south-west over northern England. Images are 256 km × 256 km. Maps produced with Cartopy and SRTM elevation data [9].

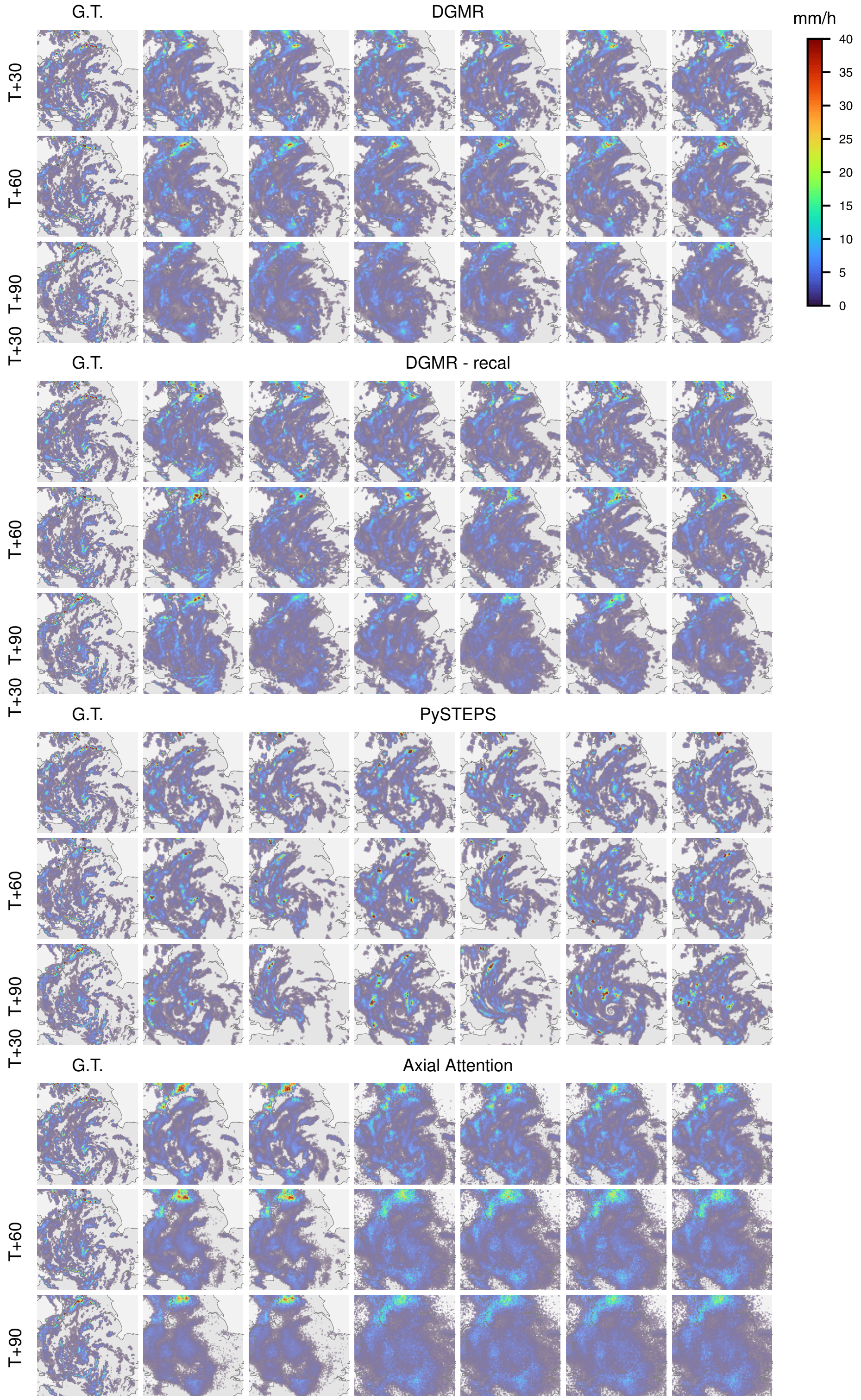

Supplementary Figure 10 | **Postage stamp plots.** Ground Truth (left) and 6 samples. The rows for each method show predictions for T+30 , T+60 , and T+90 mins lead time, for a challenging precipitation event showing a pattern of precipitation around a low pressure area which is slow moving, resulting in the cyclonic banded structures over England. Images are 256 km × 256 km. Maps produced with Cartopy and SRTM elevation data [9].

D. Axial Attention Model and MetNet Adaptations

We focused our analysis on two strong baselines, PySTEPS [10] and MetNet [11], in addition to an NWP reference baseline, and this section provides additional details of our implementation of these baseline methods. We focus here on the adaptations of Axial Attention-based methods for radar data.

MetNet [11] is a deep learning method for precipitation nowcasting that was demonstrated to outperform optical flow-based methods and numerical weather prediction models on the MRMS US dataset, and that was evaluated on prediction horizons up to 8 hours. MetNet's computation follows three steps:

- Spatial downsampling. Input frames are transformed using a convolutional network to 256-dimensional feature maps (spatial shape 64×64).
- Temporal encoding. Feature maps from consecutive input time steps are integrated using the Convolutional LSTM architecture [12] into a single 384-layer map (spatial shape 64×64).
- An Axial Attention-based aggregation [13] is applied to that map to produce a single prediction frame of spatial shape 64×64.

We made several adaptations to the MetNet algorithm[3] for use with radar data only. For disambiguation, we refer to the original implementation from [11] as MetNet and to our implementation as the Axial Attention model.

Each input and output pixel in MetNet corresponds to 0.01 deg of latitude and 0.01 deg of longitude, i.e. about 1 km (not accounting for the approximations due to the Mercator projection). The spatial extent of MetNet inputs was 1024×1024, and the method was trained to predict the central 64×64 crop. [11] assumes that precipitation moves at 60 km/hr, leaving the 480 pixel margins on the input to account for rain cloud advection over eight hours and to ensure that the model makes predictions using only data within the input frames. In our study, we operate at a scale of 1 km per pixel (UK data) and 0.01 deg per pixel (US data), making predictions at horizons up to 90 minutes. Assuming similar rain cloud maximum speed, we set margins at 96 pixels, with 256×256 inputs and 64×64 outputs.

To make the Axial Attention model comparable to other methods in this paper, we reduce the temporal extent of the input context from seven frames (covering 90 minutes with 15 minute intervals, in the MetNet implementation) down to four frames covering 20 minutes with five minute intervals. In the ablation studies in the original paper, two input frames sufficed for equivalent CSI performance at rain rate 1 mm/hr.

MetNet relies on several layers of input data: precipitation measured by radar, Geostationary Operational Environmental Satellite 16 (GOES-16), per-pixel elevation embedding, per-pixel latitude and longitude position embeddings, per-frame time embeddings. As we did not have access to geostationary data for the UK, we conducted our study using only elevation, position and time embeddings. We extracted SRTM elevation data from CGIAR[4], made available via the Google Earth Engine[5] and pre-processed it to be a single layer aligned with the OSGB coordinates of UK data or with the WGS-84 coordinates of US data. We capped elevation at 4000 m (US) and 1000 m (UK) and normalized values to be between 0 and 10 to match the scale of precipitation data. We computed positional embeddings by calculating the cosine and sine values of x and y coordinates at 4 different scales, following [4], resulting in 16 additional input layers. The paper did not specify how latitude and longitude were embedded. Similar to the original paper, we added three layers for temporal embeddings, corresponding to values of month/12, day/31 and hour/24 to the Axial Attention model.

[3] The Axial Attention implementation is available at https://github.com/google-research/google-research/tree/master/axial
[4] SRTM elevation data http://srtm.csi.cgiar.org/srtmdata/
[5] Google Earth Engine https://earthengine.google.com/

In [11], this additional context was added to account for orographic rain, differing climate and seasonality in precipitation. We evaluated the contribution of these additional 20 layers of elevation, positional and temporal embeddings to the performance of the Axial Attention model, as measured by CSI. Counter-intuitively, we observed that this additional data did not improve performance, as changes in CSI were not statistically significant. We hypothesize that, at the time scale at which nowcasting operates, the phenomena represented by these additional embeddings are modeled in the dynamics of precipitation over four input frames. MetNet applied a transformation $\tanh(\log(x + 0.01)/4)$ to input precipitation data. In our analysis, we found that transforming the input data had no effect, so we included no transformation.

MetNet is trained with lead times of 15 to 480 minutes, with a temporal resolution of 15 minutes. For each choice of prediction target, the target time is specified as an input to the model, using one-hot embeddings concatenated with input frames; models with different prediction lead times thus share the same parameters. In our case, we train MetNet to predict at lead times 5 to 90 minutes with a temporal resolution of 5 minutes (UK), and 6 to 90 minutes with a temporal resolution of 6 minutes (US).

While the Axial Attention model is trained similarly to other deterministic methods such as UNet (i.e., by using a separate loss term for each grid cell), it outputs a distribution over precipitation levels for each pixel, corresponding to the logits of a multinomial. MetNet predicted precipitation over 512 bins of width 0.2 mm/hr, from 0 to 102.4 mm/hr. Such a binning scheme does not conform to the empirical distribution of precipitation, with higher amounts of rain becoming increasingly rare, and requires a large number of network parameters to represent the output distribution. For this reason, we reduce the number of bins N and rescale target data x, starting with normalizing it to $x_n = (x - m)/(M - m)$, where the maximum of precipitation is set at $M = 60$ and the minimum at $m = 0$, followed by μ-law re-scaling to $x_\mu = \frac{\log(1+\mu * x_n)}{\log(1+\mu)}$ and finally binning $N \times x_\mu$ on integer values between 0 and $N - 1$. The number of bins $N = 32$, and the μ-law coefficient $\mu = 256$ were chosen by cross-validation. Note that for $N = 32$ and $\mu = 256$, all precipitation values above 50 mm/hr are clustered in the last bin.

Maximum likelihood training of the Axial Attention model corresponds to minimizing the cross-entropy between the predicted distribution and the ground truth categorical label. The per-pixel loss function was weighted by the square root of precipitation level, as we found this weighting helpful for predicting heavier rainfall. During evaluation, the mode of the output distribution is selected as the deterministic prediction. Note that the samples generated by MetNet have each pixel sampled independently of its neighbors. While these samples are useful for assessing the uncertainty of the per-grid-cell precipitation values, they result in noisy realizations that display grainy image features. For the purpose of human evaluation, we only presented the maximum likelihood estimate predictions (by taking the mode of the predicted distribution for each pixel) to the human judges.

Since MetNet produces the logits $y = \log p(x)$ of the probability distribution over precipitation values, we can use it to sample different realizations and estimate the model uncertainty for CRPS metrics. To do so, we compute the probability $p(x) = \frac{\exp(y_i/T)}{\sum_j \exp(y_j/T)}$ using softmax with different scaling coefficients (temperatures) T and sample from that estimated distribution. $T = 1$ corresponds to the scaling used during training, the limit case $T \longrightarrow 0$ corresponds to taking the mode. Using CRPS scores, we chose temperature $T = 0.5$ on the validation set.

As discussed in the methods, we assessed a post-processing approach for the Axial Attention model to ensure we used the strongest baseline methods we could. We developed a version of SPPT [14] using Gaussian process copulas. Using a spatially correlated noise process in this way did improve the predictive distribution of the baseline, as measured by the pooled CRPS metrics. We found a spatial correlation lengthscale of around 25km performed best, together with a temporal correlation timescale of 30 to 60 mins. The samples produced were not physically plausible from this method. When assessing these modified predictions, the Chief forecaster clarified the weakness of predictions using this type of

scheme as: "I'd have no confidence in [these schemes] and would not use them on the bench or in any form of automated output."

E. Related Work

Nowcasting is a long-standing problem in weather prediction, and our work is informed by a broad range of existing approaches and considerations. We defer to [15] for a general overview of nowcasting. In this section, we elaborate on the context of existing work and the facets of the nowcasting problem they address and that were developed in the main paper.

Deep learning architectures on lower-resolution radar. A majority of papers required the dataset to be resized to overcome computer memory limitations, and thus operated at much coarser resolutions than 1 km $\times$ 1 km. One of the earliest models was a Convolutional Long Short-Term Memory (ConvLSTM) recurrent neural network for deterministic 90-minute rain/no-rain prediction on a 480 $\times$ 480 HKO-7 dataset resized to 100 $\times$ 100 [16]. On that same resized dataset, an alternative spatio-temporal architecture with stacked RNN layers was proposed [17]. Similar PredRNN++ and PredNet architectures were used for precipitation nowcasting over Sao Paulo, Brazil and Kyoto, Japan, respectively [18, 19], although their work did not include standard metrics of performance. A "star-bridge" architecture, which uses precipitation-specific ConvLSTM layers, connections among ConvLSTM layers, and a two-threshold loss (at zero and three mm/hr), was used for deterministic prediction on a Shanghai dataset, resized from 500 $\times$ 500 to 100 $\times$ 100 [20]. An architecture combining 3DCNNs and bidirectional ConvLSTMs was evaluated on a radar dataset over Guangdong, Hong Kong, and Macao, resized from 500 $\times$ 500 to 100 $\times$ 100 for deterministic prediction with horizons of 48 minutes. A hierarchical RNN architecture was used for deterministic predictions up to 3 hours ahead on the 480 $\times$ 480 HKO-7 dataset, resized to 128 $\times$ 128 [21].

High-resolution nowcasting. Fewer works propose deterministic models that can operate at the same resolution as the underlying data and do high-resolution forecasting. These include the Convolutional Gated Recurrent Units (ConvGRU) and Trajectory GRU (TrajGRU) [16] (the latter model incorporates an optical flow module in a ConvGRU-like layer to model convective dynamics), as well as UNets with classification and regression outputs, respectively [22] and [23]. The star-bridge ConvLSTM architecture was made to work on a full-resolution 500 $\times$ 500 dataset over China [24]. Finally, [11] use a ConvLSTM encoder and axial attention decoder to perform eight hour pointwise probabilistic prediction by sliding a 64 $\times$ 64 window over a much larger area covering the United States.

Loss functions to address blurry predictions. As noted by some authors, predictions from these deterministic methods tend to be blurry. As a result, there have been attempts to introduce other alternative losses to increase prediction realism. One method is to "adversarially regularize" neural networks, which instead of creating a probabilistic model, adds discriminator losses to deterministic predictions. Most of the proposed methods generate predictions on limited spatial or temporal resolution, for example operating on 64$\times$64 data [25], or requiring to resize a 480$\times$480 dataset to 256$\times$256 and to make predictions at three time steps (30, 60, and 90 minutes) only [26]. One model used multi-elevation inputs, resized from 480$\times$480 to 128$\times$128, for 60-minute prediction, and found promising performance on low-threshold rainfall (below or above 0.5 mm/hr), but noted temporally inconsistent predictions [27]. One notable exception to the limited resolution of inputs used a UNet model with a deterministic *pix2pix* discriminator [28] on full resolution data, but its CSI performance was similar to optical flow for prediction horizons up to 60 minutes [29]. Thus far, ensemble methods have received limited attention, with the exception of recent work on training four separate TrajGRUs with the proposed loss modified

with different thresholds [30]. Computer vision-inspired losses have been used to improve sharpness in deterministic predictions [31, 32].

Preliminary GAN approaches. A probabilistic Conditional GAN model was recently proposed, which uses an autoregressive generator, conditioned on the regression output of a ConvLSTM model, and trained with adversarial losses to decrease blurriness in predictions up to 60 minutes [33]; the authors did not report accuracy results though and noted the poor performance of the model; moreover, and perhaps owing to its autoregressive generator, predictions became blurrier over time.

Related problems and further data sources. Broadening the literature survey, we notice that deep learning models have been used for super-resolution (also called *downscaling*) instead of prediction [34, 35], including GAN-based downscaling of radar images of precipitation [34, 36] and snowwater equivalent prediction from topographical data and meteorological forcings [37]. There has been some recent work on nowcasting using satellite data, which tends to have much lower spatial (10 km) and temporal (30 min) resolution, but can cover the entire globe [38–40]. Finally, machine learning techniques other than deep learning models, such as a Koopman operator [41] or Support Vector Machines (SVM) [42] have been considered for nowcasting.

Expert Forecaster Assessments. While not widely-used there is a body of work on understanding the judgements and needs of public and private sector operational meteorologists. Works in this area include early questionnaire-based work [43] and research over the last two decades [44–48].

F. Verification Metrics

For completeness, we provide further details about the per-grid-cell metrics for point predictions (F.1), ensemble predictions (F.2), pooled neighborhood metrics (F.3) and the whole-frame metric (F.4) that we relied on in the paper.

With the exception of full-frame metrics, we evaluate using the subsampled datasets described in A.1. In these datasets each example consists of $T \times h \times w$ grid cells, where $T = M + N$. Models condition on the first M context frames, and make predictions which are evaluated against the subsequent N frames, using the central $N \times 64 \times 64$ grid cells only to ensure that models are not penalized by boundary effects. We refer to these as the example's *target grid cells*. M and N are 4 and 18 for the UK data and 4 and 15 for the US data.

F.1. Per-grid-cell metrics for point predictions

Per-grid-cell metrics are computed over all target grid cells in all examples in the evaluation dataset. We index these target grid cells using a single index i, and note that a single observed grid cell may occur multiple times as a target, where it is forecast at different lead times by different overlapping examples. We will write F_i for the model's forecast for target grid cell i, and O_i for the corresponding ground truth observation. Each target grid cell i is associated with a weight $w_i = m_{radar,i} \cdot q_{n_i}^{-1}$, which is applied whenever we sum over all grid cells. Here m_{radar} is a binary mask which excludes grid cells for which no radar observation is available, and $q_{n_i}^{-1}$ is a per-example importance weight. This is the inverse of the inclusion probability q_{n_i} defined in Supplementary A.1 eq. (6), for the example n_i containing target grid cell i. It is used to implement the importance sampling scheme described in Supplementary A.1. For convenience, in the following we write $\hat{w}_i$ for the normalized weight $w_i / \sum_{i'} w_{i'}$.

F.1.1. Mean squared error (MSE) and Pearson Correlation Coefficient (PCC)

These metrics give a continuous measure of the accuracy of real-valued point predictions:

$$MSE = \sum_i \hat{w}_i (F_i - O_i)^2; \qquad PCC = \sum_i \hat{w}_i \frac{(F_i - \mu_F)}{\sigma_F} \frac{(O_i - \mu_O)}{\sigma_O}, \tag{7}$$

where $\mu_F, \mu_O, \sigma_F, \sigma_O$ are w-weighted means and standard deviations over all F_i and O_i respectively. Lower is better for MSE, and higher is better for PC.

F.1.2. Critical Success Index (CSI)

CSI [49] evaluates binary forecasts of whether or not rainfall exceeds a threshold t, for example low rain ($t = 2\,\text{mm/hr}$), medium rain ($t = 5\,\text{mm/hr}$) or heavy rain ($t = 10\,\text{mm/hr}$). It aims to give a single summary of binary classification performance that rewards both precision and recall, and is popular in the forecasting community. It is defined as

$$CSI = \frac{TP}{TP + FP + FN}.$$

We compute TP, FP and FN as sums of weights w_i over grid cells for which the forecast is respectively a true positive ($F_i \geq t, O_i \geq t$), false positive ($F_i \geq t, O_i < t$) and false negative ($F_i < t, O_i \geq t$).

CSI is a monotonic transformation of the more-widely-known f_1 classifier score (CSI $= f_1/(2 - f_1)$), and can also be viewed as a Jaccard similarity or Intersection over Union (IoU) metric computed over all target grid cells. Higher is better for CSI.

F.2. Per-grid-cell Metrics for Ensemble Predictions

Ensemble models give an ensemble of $N > 1$ forecasts for each example, which we will think of as i.i.d. samples from the predictive distribution of a probabilistic model. In our evaluation we use $N = 20$.

F.2.1. Continuous Ranked Probability Score (CRPS)

CRPS [50, 51] is a proper scoring rule [51] for univariate distributions, which we use to score the per-grid-cell marginals of a model's predictive distribution against observations. It is defined per grid cell as:

$$\mathbb{E}|F - O| - \frac{1}{2}\mathbb{E}|F - F'|,$$

where F and F' are drawn independently from the predictive distribution and O is the observation. Lower is better for CRPS. We compute unbiased estimates of CRPS using $\widehat{CRPS}_{PWM}$ from [52], with the N ensemble members as samples. These are then averaged over all grid cells as $\sum_i \hat{w}_i \widehat{CRPS}_{PWM,i}$.

F.2.2. Reliability and Sharpness Diagrams

We use reliability diagrams [53] to measure calibration for ensemble forecasts of whether rain exceeds a threshold t. The diagram plots the forecast probability against corresponding observed frequencies. For a perfectly-calibrated model the resulting curve should be aligned with the diagonal, subject to some error due to the finite-sample estimates used [54]. It is accompanied by a sharpness diagram showing the frequency with which each probability is forecast.

We estimate per-grid-cell predictive probabilities as the proportion $\hat{p}_i$ of the N ensemble forecasts which exceed the threshold. For each of the $N + 1$ possible values p for $\hat{p}_i$, we compute the frequency

with which the forecast is made (for the sharpness plot), and the observed frequency conditional on this forecast (for the reliability plot):

$$f_{pred}(p) = \sum_i \hat{w}_i \mathbb{1}[\hat{p}_i = p]; \qquad f_{obs|pred}(p) = \frac{\sum_i w_i \mathbb{1}[\hat{p}_i = p \wedge O_i \geq t]}{\sum_i w_i \mathbb{1}[\hat{p}_i = p]}.$$

F.2.3. Rank Histograms

We use rank histograms [55, 56] to measure calibration of ensemble forecasts of continuous rainfall values. For each grid cell, the N ensemble forecasts are ranked in increasing order, and the position of the observation in this ranking (from 0 to N inclusive) is computed, with tie-breaking as in [55]. We then display the frequency of these ranks in a histogram pooled over all grid cells. For perfectly-calibrated forecasts this histogram is expected to be uniform.

We compute the histogram using the implementation in PySTEPS [10]. While we exclude masked grid cells, this approach does not allow us to incorporate the importance weights as we do for other metrics. This does not change the property that a uniform histogram should be expected in the ideal case, however it will be biased to focus more on deviations from uniformity due to heavier-rainfall examples.

F.3. Pooled Neighborhood Metrics

These metrics evaluate forecasts of observations that are pooled over local $K \times K$-grid-cell neighborhoods and over a period of T timesteps. This pooling allows some credit for a forecast which gets the “big picture” correct, even if smaller-scale weather patterns or precise timings are not predicted correctly. We average over all $T \times K \times K$ spatio-temporal neighborhoods of target grid cells in all examples in the evaluation dataset, subject to a horizontal and vertical stride of $\lceil K/4 \rceil$. We weight each neighborhood j using a weight

$$w_j = \left(\prod_{i \in j} m_{radar,i} \right) \cdot q_{n_j}^{-1},$$

which is zero if any grid cell within it is masked by m_{radar}. These partially-masked neighborhoods are excluded as they do not in effect have the advertised scale of $T \times K \times K$. Here $q_{n_j}^{-1}$ is the importance weight for the containing example n_j, see eq. (6).

F.3.1. Pooled CRPS

We also compute CRPS using forecasts and observations which are pooled over local neighborhoods, using both average and max pooling. These are weighted using the neighborhood weights w_j. Pooled CRPS evaluates more than just the per-grid-cell marginals of the predictive distribution, requiring some modeling of the dependence between nearby grid cells to accurately forecast the marginals for pooled values. It can be motivated as a crude proxy for performance on tasks such as flood prediction, which require probabilistic forecasts of aggregate rainfall or maximum rainfall over broader catchment areas.

F.3.2. Economic value and cost-loss ratio decision

We use the decision-analytic model from [57] to evaluate the economic value of binary forecasts of weather events. The events we consider are average rainfall exceeding a given threshold, over spatio-temporal neighborhoods of a given scale and duration.

This evaluation uses a cost-loss ratio decision model, where for each weather event we must choose whether or not to take a precautionary action. If we take precautions we incur a fixed cost C; if we don’t take precautions and the weather event occurs, we incur a loss L. For a weather event with climatological

probability p_c the climatological mean expense is $E_c = \min(C, p_c L)$, whereas the expense of a perfect forecast is $E_p = p_c C$. The expense of a forecast E_f and its value V relative to these baselines are:

$$E_f = \frac{(TP + FP) * C + FN * L}{TP + FP + TN + FN}; \qquad V = \frac{E_f - E_c}{E_p - E_c}.$$

where TP, FP, TN, FN are sums of weights for neighborhoods in which the forecast is respectively a true positive, false positive, true negative or false negative, and the forecast is made by applying a decision threshold to the proportion of ensemble members in which each event occurs.

Note that V depends on C and L only via the cost-loss ratio C/L. We plot the value V for a range of cost-loss ratios between 0 and 1, choosing a decision threshold separately for each cost-loss ratio to maximize the value achieved.

In this analysis, we are interested in having high value for lower cost-loss ratios where incurring a loss is more serious, while also having a generally higher area under the cost-loss curve.

F.4. Radially-averaged power spectral density (PSD)

We report radially-averaged power spectral density [58, 59], using the implementation from PySTEPS [10]. This measures how power is distributed across a range of spatial frequencies in each model's forecasts, compared with observations.

References

1. Kahn, H. *Use of different Monte Carlo sampling techniques* (Rand Corporation, 1955).
2. Zhang, J. *et al.* Multi-radar multi-sensor (MRMS) quantitative precipitation estimation: Initial operating capabilities. *Bull. Amer. Meteorol. Soc.* **97**, 621–638 (2016).
3. Smith, T. M. *et al.* Multi-radar multi-sensor (MRMS) severe weather and aviation products: Initial operating capabilities. *Bull. Amer. Meteorol. Soc.* **97**, 1617–1630 (2016).
4. Vaswani, A. *et al.* Attention is all you need. In *Advances in Neural Information Processing Systems*, vol. 30, 5998–6008 (2017).
5. Zhang, H., Goodfellow, I., Metaxas, D. & Odena, A. Self-attention generative adversarial networks. In *International Conference on Machine Learning*, vol. 36, 7354–7363 (PMLR, 2019).
6. Luc, P. *et al.* Transformation-based Adversarial Video Prediction on Large-Scale Data. *Preprint at https://arxiv.org/abs/2003.04035* (2020). 2003.04035.
7. Sun, J. *et al.* Use of NWP for nowcasting convective precipitation: Recent progress and challenges. *Bull. Amer. Meteorol. Soc.* **95**, 409–426 (2014).
8. Bush, M. *et al.* The first Met Office unified model–JULES regional atmosphere and land configuration, RAL1. *Geosci. Mod. Dev.* **13**, 1999–2029 (2020).
9. A., J., Reuter, H., Nelson, A. & Guevara, E. Hole-filled seamless SRTM data V4, international centre for tropical agriculture (ciat) (2008). URL https://srtm.csi.cgiar.org.
10. Pulkkinen, S. *et al.* PySTEPS: An open-source python library for probabilistic precipitation nowcasting (v1. 0). *Geosci. Mod. Dev.* **12**, 4185–4219 (2019).

11. Sønderby, C. K. *et al.* MetNet: A neural weather model for precipitation forecasting. *Preprint at https://arxiv.org/abs/2003.12140* (2020).

12. Xingjian, S. *et al.* Convolutional LSTM network: A machine learning approach for precipitation nowcasting. In *Advances in Neural Information Processing Systems*, vol. 28, 802–810 (2015).

13. Ho, J., Kalchbrenner, N., Weissenborn, D. & Salimans, T. Axial attention in multidimensional transformers. *Preprint at https://arxiv.org/abs/1912.12180* (2019).

14. Palmer, T. *et al.* Stochastic parametrization and model uncertainty. *ECMWF Technical Memoranda* (2009).

15. Prudden, R. *et al.* A review of radar-based nowcasting of precipitation and applicable machine learning techniques. *Preprint at https://arxiv.org/abs/2005.04988* (2020).

16. Shi, X. *et al.* Deep learning for precipitation nowcasting: A benchmark and a new model. In *Advances in Neural Information Processing Systems*, vol. 30, 5617–5627 (2017).

17. Wang, Y., Long, M., Wang, J., Gao, Z. & Philip, S. Y. PredRNN: Recurrent neural networks for predictive learning using spatiotemporal LSTMs. In *Advances in Neural Information Processing Systems*, vol. 30, 879–888 (2017).

18. Bonnet, S. M., Evsukoff, A. & Morales Rodriguez, C. A. Precipitation nowcasting with weather radar images and deep learning in São Paulo, Brasil. *Atmosphere* **11**, 1157 (2020).

19. Marrocu, M. & Massidda, L. Performance comparison between deep learning and optical flow-based techniques for nowcast precipitation from radar images. *Forecasting* **2**, 194–210 (2020).

20. Cao, Y. *et al.* Precipitation nowcasting with star-bridge networks. *Preprint at https://arxiv.org/abs/1907.08069* (2019).

21. Jing, J., Li, Q., Peng, X., Ma, Q. & Tang, S. HPRNN: A hierarchical sequence prediction model for long-term weather radar echo extrapolation. In *IEEE International Conference on Acoustics, Speech and Signal Processing*, 4142–4146 (IEEE, 2020).

22. Agrawal, S. *et al.* Machine learning for precipitation nowcasting from radar images. *Preprint at https://arxiv.org/abs/1912.12132* (2019).

23. Ayzel, G., Scheffer, T. & Heistermann, M. Rainnet v1.0: A convolutional neural network for radar-based precipitation nowcasting. *Geosci. Mod. Dev.* **13**, 2631–2644 (2020).

24. Chen, S. *et al.* Strong spatiotemporal radar echo nowcasting combining 3DCNN and bi-directional convolutional LSTM. *Atmosphere* **11**, 569 (2020).

25. Singh, S., Sarkar, S. & Mitra, P. A deep learning based approach with adversarial regularization for Doppler weather radar ECHO prediction. In *IEEE International Geoscience and Remote Sensing Symposium (IGARSS)*, 5205–5208 (IEEE, 2017).

26. Jing, J. *et al.* AENN: A generative adversarial neural network for weather radar echo extrapolation. *International Archives of the Photogrammetry, Remote Sensing and Spatial Information Sciences (ISPRS)* **42**, 89–94 (2019).

27. Jing, J., Li, Q. & Peng, X. MLC-LSTM: Exploiting the spatiotemporal correlation between multi-level weather radar echoes for echo sequence extrapolation. *Sensors* **19**, 3988 (2019).

28. Isola, P., Zhu, J.-Y., Zhou, T. & Efros, A. A. Image-to-image translation with conditional adversarial networks. In *Conference on Computer Vision and Pattern Recognition*, 1125–1134 (IEEE, 2017).

29. Veillette, M., Samsi, S. & Mattioli, C. SEVIR: A storm event imagery dataset for deep learning applications in radar and satellite meteorology. In *Advances in Neural Information Processing Systems*, vol. 33 (2020).

30. Franch, G. *et al.* Precipitation nowcasting with orographic enhanced stacked generalization: Improving deep learning predictions on extreme events. *Atmosphere* **11**, 267 (2020). URL http://dx.doi.org/10.3390/atmos11030267.

31. Tran, Q.-K. & Song, S.-k. Computer vision in precipitation nowcasting: Applying image quality assessment metrics for training deep neural networks. *Atmosphere* **10**, 244 (2019).

32. Tran, Q.-K. & Song, S.-k. Multi-channel weather radar echo extrapolation with convolutional recurrent neural networks. *Remote Sensing* **11**, 2303 (2019).

33. Liu, H.-B. & Lee, I. MPL-GAN: Toward realistic meteorological predictive learning using conditional GAN. *IEEE Access* **8**, 93179–93186 (2020).

34. Leinonen, J., Nerini, D. & Berne, A. Stochastic super-resolution for downscaling time-evolving atmospheric fields with a generative adversarial network. *IEEE Transactions on Geoscience and Remote Sensing* 1–13 (2020).

35. Adewoyin, R., Dueben, P., Watson, P., He, Y. & Dutta, R. Tru-net: A deep learning approach to high resolution prediction of rainfall. *Preprint at https://arxiv.org/abs/2008.09090* (2020).

36. Chen, H., Zhang, X., Liu, Y. & Zeng, Q. Generative adversarial networks capabilities for super-resolution reconstruction of weather radar echo images. *Atmosphere* **10**, 555 (2019).

37. Manepalli, A., Albert, A., Rhoades, A., Feldman, D. & Jones, A. D. Emulating numeric hydroclimate models with physics-informed cGANs. In *AGU Fall Meeting 2019* (AGU, 2019).

38. Kumar, A., Islam, T., Sekimoto, Y., Mattmann, C. & Wilson, B. Convcast: An embedded convolutional LSTM based architecture for precipitation nowcasting using satellite data. *Plos one* **15**, e0230114 (2020).

39. Zantedeschi, V. *et al.* Towards data-driven physics-informed global precipitation forecasting from satellite imagery. In *Proceedings of the AI for Earth Sciences Workshop at NeurIPS* (2020).

40. de Witt, C. S. *et al.* Rainbench: Towards global precipitation forecasting from satellite imagery. *Preprint at https://arxiv.org/abs/1912.12180* (2020).

41. Zheng, S., Miyamoto, T., Iwanami, K., Shimizu, S. & Kato, R. Hybrid scheme of kinematic analysis and Lagrangian Koopman operator analysis for short-term precipitation forecasting. *Preprint at https://arxiv.org/abs/2006.02064* (2020).

42. Han, L. *et al.* A machine learning nowcasting method based on real-time reanalysis data. *Journal of Geophysical Research: Atmospheres* **122**, 4038–4051 (2017).

43. Murphy, A. H. & Winkler, R. L. Forecasters and probability forecasts: The responses to a questionnaire. *Bull. Amer. Meteorol. Soc.* **52**, 158–166 (1971).

44. Doswell III, C. A. Weather forecasting by humans—heuristics and decision making. *Weather Forecast.* **19**, 1115–1126 (2004).

45. Demeritt, D., Nobert, S., Cloke, H. & Pappenberger, F. Challenges in communicating and using ensembles in operational flood forecasting. *Meteorological applications* **17**, 209–222 (2010).

46. Evans, C., Van Dyke, D. F. & Lericos, T. How do forecasters utilize output from a convection-permitting ensemble forecast system? case study of a high-impact precipitation event. *Weather Forecast.* **29**, 466–486 (2014).

47. Demuth, J. L. *et al.* Recommendations for developing useful and usable convection-allowing model ensemble information for nws forecasters. *Weather Forecast.* **35**, 1381–1406 (2020).

48. Cintineo, J. L., Pavolonis, M. J., Sieglaff, J. M., Cronce, L. & Brunner, J. Noaa probsevere v2. 0—probhail, probwind, and probtor. *Weather Forecast.* **35**, 1523–1543 (2020).

49. Schaefer, J. T. The critical success index as an indicator of warning skill. *Weather Forecast.* **5**, 570–575 (1990).

50. Matheson, J. E. & Winkler, R. L. Scoring rules for continuous probability distributions. *Management science* **22**, 1087–1096 (1976).

51. Gneiting, T. & Raftery, A. E. Strictly proper scoring rules, prediction, and estimation. *J. Am. Stat. Assoc.* **102**, 359–378 (2007).

52. Zamo, M. & Naveau, P. Estimation of the continuous ranked probability score with limited information and applications to ensemble weather forecasts. *Mathematical Geosciences* **50**, 209–234 (2018).

53. Murphy, A. H. & Winkler, R. L. Reliability of subjective probability forecasts of precipitation and temperature. *Journal of the Royal Statistical Society: Series C (Applied Statistics)* **26**, 41–47 (1977).

54. Bröcker, J. & Smith, L. A. Increasing the reliability of reliability diagrams. *Weather Forecast.* **22**, 651–661 (2007).

55. Hamill, T. M. & Colucci, S. J. Verification of Eta–RSM short-range ensemble forecasts. *Mon. Weather Rev.* **125**, 1312–1327 (1997).

56. Hamill, T. M. Interpretation of rank histograms for verifying ensemble forecasts. *Mon. Weather Rev.* **129**, 550–560 (2001).

57. Richardson, D. S. Skill and relative economic value of the ECMWF ensemble prediction system. *Quart. J. Roy. Meteorol. Soc.* **126**, 649–667 (2000).

58. Harris, D., Foufoula-Georgiou, E., Droegemeier, K. K. & Levit, J. J. Multiscale statistical properties of a high-resolution precipitation forecast. *J. Hydrol.* **2**, 406–418 (2001).

59. Sinclair, S. & Pegram, G. Empirical mode decomposition in 2-D space and time: A tool for space-time rainfall analysis and nowcasting. *Hydrol. and Earth Sys. Sci.* **9**, 127–137 (2005).
